# Chemical Sensing Applications of ZnO Nanomaterials

**DOI:** 10.3390/ma11020287

**Published:** 2018-02-12

**Authors:** Savita Chaudhary, Ahmad Umar, K. K. Bhasin, Sotirios Baskoutas

**Affiliations:** 1 Department of Chemistry and Centre of Advanced Studies in Chemistry, Panjab University, Chandigarh 160014, India; sav66hooda@gmail.com; 2 Department of Chemistry, College of Science and Arts, Najran University, Najran 11001, Saudi Arabia; 3 Promising Centre for Sensors and Electronic Devices (PCSED), Najran University, Najran 11001, Saudi Arabia; 4 Department of Materials Science, University of Patras, Patras GR 26504, Greece; bask@upatras.gr

**Keywords:** zinc oxide, synthesis, chemical sensing, sensitivity, selectivity, morphology

## Abstract

Recent advancement in nanoscience and nanotechnology has witnessed numerous triumphs of zinc oxide (ZnO) nanomaterials due to their various exotic and multifunctional properties and wide applications. As a remarkable and functional material, ZnO has attracted extensive scientific and technological attention, as it combines different properties such as high specific surface area, biocompatibility, electrochemical activities, chemical and photochemical stability, high-electron communicating features, non-toxicity, ease of syntheses, and so on. Because of its various interesting properties, ZnO nanomaterials have been used for various applications ranging from electronics to optoelectronics, sensing to biomedical and environmental applications. Further, due to the high electrochemical activities and electron communication features, ZnO nanomaterials are considered as excellent candidates for electrochemical sensors. The present review meticulously introduces the current advancements of ZnO nanomaterial-based chemical sensors. Various operational factors such as the effect of size, morphologies, compositions and their respective working mechanisms along with the selectivity, sensitivity, detection limit, stability, etc., are discussed in this article.

## 1. Introduction

The rapid growth of industries and frequent use of chemicals in textile, pharmaceutical, food and automobile industries have contributed to a major threat to the survival of living beings on the Earth [1,2,3]. The emission of harmful toxins from automobile exhaust and factory outlets has become a major source of environmental pollution. Therefore, an authentic means for the effectual recognition of harmful chemicals by using chemical and biological sensors is in urgent need of the present [4,5]. Of all available types of semiconductor sensors for different types of chemicals and biological toxins, zinc oxide (ZnO)-based sensors have gained extensive attention around the world. The presence of a good response rate towards the chemical toxins with outstanding selectivity and sensitivity makes it one of the most significant materials for preparing low cost sensors [6]. The existence of a diverse range of morphologies such as nanorods, wires, needles, ellipsoids, urchins, helices, combs, flowers and disk shapes of ZnO materials has provided good control over the surface to volume ratio for the prepared nanomaterials and enhances their utility in sensing devices [7].

ZnO nanomaterials have been widely considered for their significant applications in different classes of nanoscale serviceable tools used in chemical, medical, diagnostics, food and nationwide defense-based equipment [8]. Being an n-type semiconductor with a wide band gap of 3.37 eV and a large exciton binding energy of 60 meV, the electron mobility in ZnO nanomaterials is enhanced. The existence of a high, photoelectric reaction with an admirable chemical and thermal stability makes ZnO nanomaterials among the potential contenders for the preparation of effective chemical and biological sensors [9]. In addition, ZnO nanomaterials have the advantages of a low cost of production, a harmless nature and a simple mode of large-scale production [10]. Furthermore, ZnO nanomaterials are chemo-resistive in nature, and their sensing aptitude is principally restricted by the change in the chemical signal when the respective analyte molecules encounter its exterior surface [11].

In recent years, much attention has been given to ZnO-based nanostructures for sensing. The presence of the numerous properties of ZnO has been utilized for the development of effective and highly selective sensors. For instance, gas sensors are prepared due to the variations in the conductance with the reversible chemisorption process of reactive gases on the surface of ZnO [12]. The non-lethal nature of ZnO nanoparticles has been used for the generation of effective biosensors [13]. Although to date, a large number of literature works has been produced for the fabrication of different types of sensing devices based on ZnO nanostructures, the challenge of an effective and selective sensing was still not discussed in detail [14,15].

The present review meticulously introduces the current advancements of ZnO nanostructure-supported sensors with the main emphasis on chemical and biosensors for different analytes. The different types of operational factors such as the effect of size, morphology and the respective working mechanisms of nano-ZnO-based sensors, along with their selectivity and sensitivity behavior will be considered in detail.

## 2. ZnO Nanomaterials for Sensing Applications

The presence of high surface area, the biocompatible nature, thermal stability, wide band gap and superior response towards the photoelectric reaction makes ZnO nanomaterials among the candidates for the manufacture of useful chemical and biological sensors for a diverse range of moieties [16]. The existence of a minute size range with great variations in the surface to volume ratio makes them very effective for the adsorption of harmful analytes on the exterior surface of particles. The higher surface area of ZnO has also provided an additional amount of surface active sites for the analytes [17]. The presence of more surface atoms has generated the active sensing layer for the materials to be sensed from the surrounding environment. Moreover, the small grain size, i.e., as small as the depth of the space-charge layer in the ZnO-based nanostructure, has greatly controlled the sensing response to different types of toxins [18]. Due to such a behavioral aspect, the response rate of the ZnO-based sensor has been exponentially amplified with the reduction of the size of the particles formed. The size variations in the formed materials also influenced the van der Waals force of the particles. These forces were decreased with the reduction in the size of ZnO-based nanostructures and influenced the sensing aptitude. In addition to the size, the surface morphology of the particles has also influenced the sensing behavior of ZnO-based sensors [19]. These surface morphologies of the particles have a direct influence on the number of surface defects and the porosity ZnO nanostructures. These factors have a direct influence on the electrochemical sensing aptitude for various types of biological, as well as chemical species. The existence of different types of shapes of ZnO nanostructures has provided a diverse range of spatial structures and specific areas for the particles formed. These particles have also provided a diverse range of capabilities for the circulation of analytes during adsorption-desorption of different types of moieties [20]. Furthermore, these different types of morphologies have a direct influence on the amount of surface defects, involving the concentration of oxygen vacancies to modulate the conductivity of ZnO nanoparticles, which is quite essential for the detection of chemical analytes by using electrochemical sensing [21]. Theses factor have further affected the surface sensing aptitude of the particles formed and decreased the required temperature for the detection of the analyte. For instance, one-dimensional nanostructures of ZnO possessed an excessive amount of electrical conductivity as compared to other structures. Their superior electron mobility encouraged the effectual division among the electrons and holes in the nanostructures formed and helped to decrease the amount of generated resistance in the particles formed. Under normal atmospheric conditions, oxygen molecules from the atmosphere have the ability to be adsorbed on the surface of ZnO and then converted into reactive oxygen species by taking the electrons from the conduction band of ZnO nanomaterials, escorting them to the generation of the surface depletion layer on the surface of the particles and, thus, enhancing the sensor resistance with respect to different analytes [22]. When reactive analytes come near the surface of ZnO, these reactive oxygen molecules will interact with them and release the ensnared electrons towards the conduction band and producing a change in the signal [23]. In addition, the sensing aptitude of ZnO-based chemical and biological sensors is chiefly reliant on the working temperature ranges, which further modulate the reaction kinetics, the conductive nature and the electron mobility of these nanomaterials [24]. The ZnO-based nano-/micro-structures also possess high crystal quality with periodical structures in their geometry and smooth exterior surfaces with roughly the same wavelength-level size, which can be utilized for the generation of optical microcavities on the surface of nanomaterials. These microcavities provide an intermediate path for the sensing of the external analyte. Further, the interactions of the external materials can be accurately regarded in the recommended manner, which is very supportive of the analysis of the external moieties [25]. Using the external templating agents and prescribed assembly of ZnO materials, these nanomaterials can be simply accumulated on the surface of the electrode for the manufacturing of sensing electrodes for the effective and highly receptive detection of harmful heavy metal ions and gases [26,27]. The selectivity of the sensor is also modulated by using the incorporation of functional groups on the surface of ZnO nanostructures. The surface functionalization has the ability to improve the surface to volume ration and has provided extra sites for the adsorption of analyte during the sensing process. The presence of piezoelectric properties in the ZnO nanostructures has provided new dimensions for the generation of pressure sensors. Therefore, it is not wrong to say that these ZnO nanostructures have resulted in different devices with very high sensing capabilities. However, the question of a highly selective reaction still remains a great challenge. Therefore, the current review aims to present the current achievements of ZnO nanostructure-based chemical and biological sensors.

## 3. Chemical Sensing Applications of ZnO Nanomaterials

The estimation of the harmful chemicals present in the ecosystem is one of the prime issues to keep our environment hygienic and secure. Therefore, engineered materials for the preparation of chemical sensors capable of recognizing harmful toxins has received substantial attention from the scientific community. For a particular kind of chemical sensor, one can recognize a transformation in electrical or optical signals as an effect of chemical and physical associations with external toxins. In general, these chemical sensors [28] are very constructive in the fabrication of different types of security-based devices, where one can estimate any kind of leakage of toxins. It is well-known that ZnO nanoparticles are employed as in chemical sensors due to their extensive range of stability under thermal and chemical variations. The alteration in resistance due to the presence of external adsorbed surface species is mainly attributable to the oxygen vacancy in these ZnO materials [28].

In early investigations, chemical sensors were based on the inherent properties of the electrode materials used. As a result, studies were mostly centered on the choice of an appropriate material for the development of effective sensors, and the production of novel materials for sensing became a main focus of research. With the latest progress in the area of nanoscience, the above-mentioned paradigm has totally changed. Currently, the utilization of these engineered materials has provided a better substitute for conventional electrode materials, giving more control over the sensitivity, selectivity, and stability of the developed sensors [29]. The regular types of ZnO-based nanostructure with uniform size distribution can markedly advance the detection ability, as evaluated against disordered structures of different materials. These behavioral variations were explained due to the high crystalline nature of ZnO particles with large contact area. ZnO nanostructures have more catalytic sites and better control over the electron transfer resistance in the presence of external analytes [30]. Therefore, the internal properties of ZnO-based nanostructures are considered as one of the potential factors for the effective performance of sensors.

### 3.1. Hydrazine and Phenyl Hydrazine Chemical Sensor

**Figure 1:**
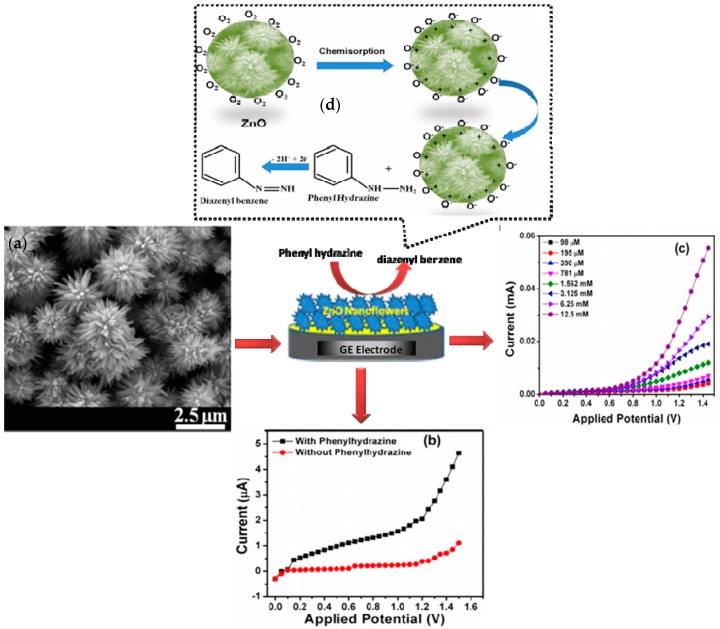
(**a**) Electrochemical measurement of fabricated phenyl hydrazine chemical sensor by using ZnO nano-urchins. (**b**,**c**) current-voltage (I–V) response in the presence and absence of phenyl hydrazine by employing the modified GCE in 10 mL, 0.1 M phosphate-buffered saline (PBS) solution. (**d**) Schematic mechanism of sensing. Adapted figure from [35] with permission from copyright, (2015), Elsevier.

Hydrazine and phenyl hydrazine are chemicals which are mainly employed in textiles, pesticides, aerospace fuel, and pharmaceutical industries. The excessive utilization of these chemicals has created the problem of their discharge in the surrounding environment, producing toxic effects in living beings [31]. Their adverse effects at minute concentrations have produced undesirable consequences for flora and fauna. Scientists have prepared a large number of metal oxide-based chemical sensors due to their potential properties of easy preparation with low processing costs and eco-friendly nature [32]. Among the different types of metal oxide materials, ZnO is extensively employed for the fabrication of sensors for perilous chemicals. This is due to the conductive nature of the ZnO nanostructure and its high chemical and thermal strength under the operation conditions of the developed sensor. For instance, Ameen et al. used vertically aligned nanorods of ZnO as a modification of the electrode surface for the estimation of hydrazine [33]. Additionally, Ibrahim et al. prepared a highly sensitive and selective electrochemical chemical sensor for phenyl hydrazine by using Ag-doped ZnO nanoflowers [34]. Umar et al. have reported the application of nano-urchins of ZnO particles for preparing sensory electrodes for the recognition of phenyl hydrazine by using the current-voltage (I–V) technique. [35]. The preparation of the nano-urchins was achieved by using the hydrothermal method at low-temperature conditions of ~165 °C. The urchin structures of ZnO are mainly generated by the gathering of numerous nanoneedles which are begun from a distinct center. These nanoneedles display sharp tips with broad bottoms. The characteristic diameter of each nanoneedle at their tips and bases were 45 ± 10 nm and 180 ± 20 nm, respectively. It was also found that the ZnO-based nano-urchins were independently developed with high density under normal synthetic conditions with a size ranging to 2 µm. The electrode was mainly prepared by using a slurry of nano-urchin-shaped ZnO particles on the surface of a glassy carbon electrode with a surface area of 0.0316 cm^2^ (Figure 1). The respective measurements of the current response were done from 0.0 to +1.5 V with the time delay and response times of 1.0 and 10.0 s, respectively, for the developed sensor in phosphate buffer of 0.1 M. The sensing performance of the ZnO was tested against the wide range of concentration of phenyl hydrazine concentration ranging from 98 µM to 25 mM. The estimation of phenyl hydrazine with ZnO-based nano-urchins can be achieved due to the oxidation and reduction characters of ZnO particles. The presence of ZnO nanostructures has shown the enhancement of current which is mainly associated with the excellent electro-catalytic performance and superior sensitivity of as-prepared ZnO nano-urchins for the estimation of phenyl hydrazine.

**Figure 2:**
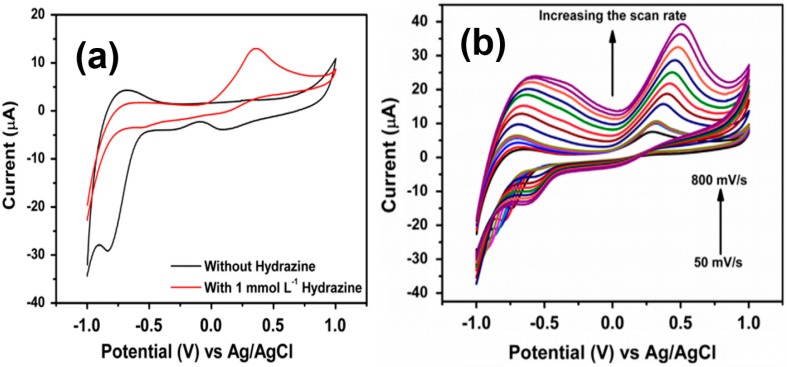
(**a**) Typical cyclic voltammetry (CV) sweep curve for Ag-ZnO nanoellipsoids/Au modified electrode with and without 11.0 mmol·L^−1^ hydrazine in 0.1 mol·L^−1^ phosphate buffer solution (PBS; pH ~ 7) at scan rate of 100 mV/s; (**b**) CV sweep curves at different scan rates (50, 60, 70, 80, 90, 100, 200, 300, 400, 500, 600, 700, and 800 mV/s) of Ag-ZnO nanoellipsoids/Au modified electrode. Adapted figure from [37] with permission from copyright (2015), Elsevier.

The adsorbed oxygen molecules play an imperative function in the sensing process. At the beginning, the molecules of O_2_ are mainly adsorbed in their ionic sate on the surface of ZnO nano-urchins (Figure 1a). These adsorbed oxygen ions have the tendency to eradicate the available electrons in the conduction band. The resultant depletion region possesses a low conductivity range close to the surface of ZnO, which significantly reduces the overall conductivity of the formed ZnO. The as-formed ionic oxygenated species has the ability to react with phenyl hydrazine to generate molecules of diazenyl benzene (Figure 1d). Then, the electrons are sent back to the respective conduction band of ZnO and there is a significant enhancement in current (Figure 1b,c). Similarly, composites of ZnO particles with SiO_2_ have also been utilized for the detection of phenyl hydrazine by using current-potential (I–V) measurements. The detection limit of the formed sensor was found to be 1.42 μM with sensitivity values reaching 10.80 μA·cm^−2^·mM^−1^ as a result of good adsorption ability and large surface area of the prepared sensor [36]. The main purpose of incorporating of SiO_2_ with ZnO nanoparticles is to enhance the stability of the formed particles during the sensing runs. The obtained composite was reusable up to five times without any decrease in the sensitivity value. In addition, there was no decline in the I–V characteristics after three months of storage, showing the long-term stability of the formed sensor. Kumar and coworkers [37] have used Ag doping on the surface of nanoellipsoids of ZnO particles for the development of effective chemical sensors for hydrazine. The presence of Ag ion doping has the ability to enhance the interfacial charge transfer ability of the formed ZnO particles. The metal ion doping has a direct influence over the absorption characteristics of ZnO particles, and hence modifies their catalytic behavior in aqueous media. The presence of Ag in the lattice of ZnO particles has a direct influence on the light absorption ability of ZnO nanomaterial, improving its sensing aptitude towards hydrazine molecules. Figure 2 displays the respective representation of cyclic voltammetry (CV) data showing the effect of hydrazine in the presence and absence of Ag-doped ZnO nanoellipsoids at a scan rate of 100 mV/s.

**Figure 3:**
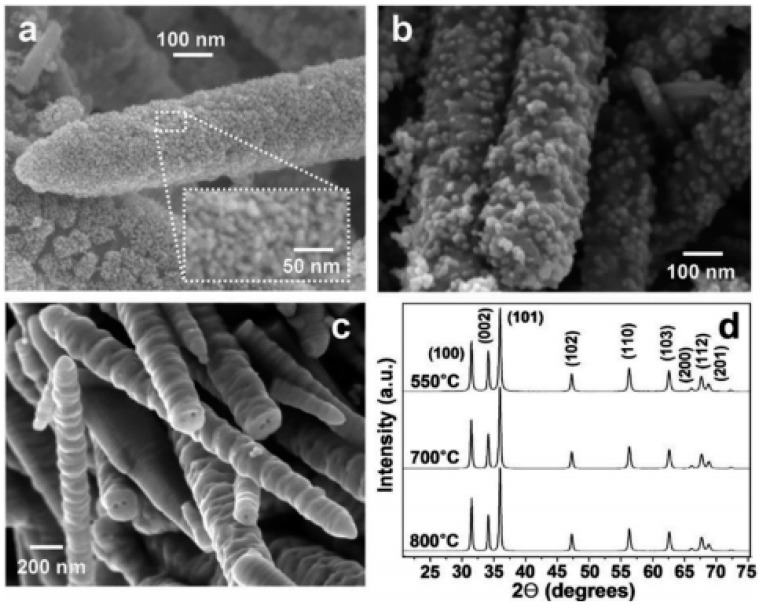

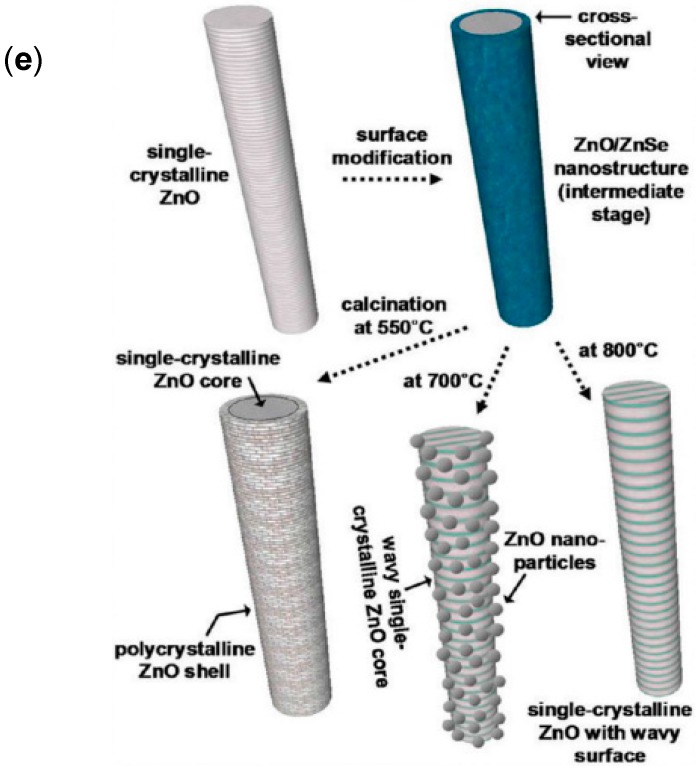
SEM images showing the effect of calcinations on ZnO particles in air for 3 h at (**a**) 550 °C; (**b**) 700 °C; or (**c**) 800 °C; (**d**) XRD patterns of the powders after calcination of the powders in air for 3 h; (**e**) Schematic illustration of the procedure for achieving surface texturing of a single-crystalline ZnO nanorod. Figure adapted from [38] with permission from copyright (2011), American Chemical Society (Washington, DC, USA).

The existence of a clear oxidation peak at 0.357 V was observed for 1.0 mmol·L^−1^ of hydrazine molecules in buffer solution. The peak also displayed the significant enhancement in height in presence of ZnO molecules. This aspect was mainly due to the fast exchange of electrons between the nanomaterials and analyte molecules. Moreover, the anodic peak current showed significant enhancement with the variation of scan rate from 50 to 800 mV/s. There was a peculiar shift in the peak potential toward the positive side with varying scan rates. The effect of annealing temperature has also been used to investigate the performance of prepared chemical sensors for phenyl hydrazine molecules. By varying the temperature, it was found that the obtained size and crystalline nature of ZnO particles was varied (Figure 3). There was a significant effect on the surface of the formed particles, and their corresponding electron transport ability during the sensing was affected by the change in the particles’ surface area [38].

The electron exchange rate and electrocatalytic oxidation characteristics of ZnO particles were affected with the variations of temperature [39]. Zhao et al. have used nanowires of ZnO for the preparation of a wide-linear range sensory device for the detection of hydrazine [40]. Ni et al. and Fang et al. have employed hierarchical micro/nanoarchitectures of ZnO and carbon nanotube-modified nanoflowers of ZnO for the detection of hydrazine [41,42]. The microstructures were mainly prepared by using the structure-directing group (i.e., hexamethylenetetramine) during the synthesis of ZnO nanomaterials. In addition, the presence of NH_3_·H_2_O has played a critical role during the generation of hierarchical structures of ZnO-based micro/nanoarchitectures. It was found that when there was no addition of NH_3_·H_2_O molecules, the formation of irregular nanosheets was observed. Regarding the addition of NH_3_·H_2_O in the reaction media, the respective columnar-shaped hexagonal microcrystals of ZnO were formed with high density. Moreover, electrochemical testing results have shown that flower-shaped particles of ZnO possessed more potential to support the oxidation process of hydrazine as compared to other structures. The only disadvantage of the formed sensor was the poor selectivity towards hydrazine. Nanocones of ZnO particles were also used for the selective detection of hydrazine with a sensitivity of 50 × 10^4^ µA·µM^−1^ cm^−2^ and detection limit 0.01 mM. The oxidation peak for hydrazine was observed at 424 mV with the peak current value of 380 mA. The C-V data clearly demonstrated highly responsive behavior of ZnO particles for hydrazine, and hence substantiate that the nanocones of ZnO are quite efficient in the electron transportation between the particles and external analyte [43]. Liu et al. used pristine nanorods of ZnO for modifying the surface of an alloy-based electrode for the sensing of hydrazine molecules [44]. The fabrication of the sensor was mainly achieved by the simple immersion-calcination process on the surface of inert alloy, and was employed for the preparation of a working electrode to build an effective sensor for hydrazine. The enhancement of electrocatalytic activity was mainly explained on the basis of synergistic behavior among the inert layer of carbon and ZnO nanorods. The electron transport rate was found to be significantly affected along the growth direction of prepared nanoparticles. For instance, these nanorods have the ability to grow in a one-dimensional (1D) pathway and possessed a greater electrochemical sensing aptitude along the length direction. It was observed that these rods had the tendency to align directly on the surface of electrode substrates; the rate of electron transport was affected, and hydrazine sensing was found to be improved. These aligned nanorods/nanowires can provide abundant “nanoelectrodes” on the surface to improve the electrochemical action. These rods provide more surface area for the transportation of electrons, and hence affect the sensing ability of the electrodes. Moreover, these ZnO nanorods have the ability to construct binder-free, stable, and highly-active chemical sensors for hydrazine. The incorporation of metallic silver with vertically-aligned zinc oxide nanorods has the tendency to modulate the electron transport and hence affect the sensing performance of the developed sensor [45]. The obtained sensitivity for the Ag@ZnO nanorods was found to be 105.5 µA·µM^−1^·cm^−2^, with a working concentration of 98.6 µM. The sensor had the ability to detect concentrations as low as 0.005 µM. The higher stability, its reproducible nature, and its selectivity for hydrazine make it more effective for the detection of hydrazine. Spiked samples from different water sources have also shown effective results for the estimation of hydrazine with the help of Ag@ZnO nanorods. The performance of the formed sensor was mainly explained due to the presence of directly growing nanostructures of ZnO particles, which further affected the kinetics for electron transference. Thus, the formed sensor has future prospective roles in binder-free, economical, and highly stable and reproducible sensors for harmful analytes.

On the other hand, electrodeposition methods have also been used for the fabrication of ZnO nanostructures on the surface of gold or glass electrodes for the selective sensing of hydrazine molecules. These methods have direct control over the morphology and size of the formed nanoparticles. The respective surface area of the prepared nanoparticles was also modulated by using the electrodeposition process. The variations in the deposition parameters included changes in the concentration of external agent, the potential value used, as well as the applied current, and directly influenced the deposition layer of ZnO particles. The electrocatalytic activity of the formed nanoparticles was also varied with the concentration of hydrazine moieties, and displayed a different sensitivity and detection limit than other morphologies (Table 1).

Recently, Zhang et al. used a combination of zeolitic imidazolate framework-8 (ZIF-8)-derived N-doped carbon film-immobilized gold nanoparticles (AuNPs) on a ZnO jungle for the estimation of hydrazine [56]. The in-situ oxidation was mainly responsible for the synthesis of N-doped carbon film-coated ZnO particles used for the sensor fabrication. The jungle of ZnO structures was mainly formed by nanorods of ZnO particles generated by the electrodeposition process. The presence of Au in combination with ZnO provided better control over the catalytic activity of hydrazine and provided a distinguished current peak at 0.66 V with a current of 6.19 µA. The detection of hydrazine mainly involves the transference of four electrons in the system [57]. The low-temperature-based synthesis of ZnO particles with high aspect ratio has also been used for the fabrication of chemical sensors for hydrazine molecules with the ultra-high sensitivity of ∼97.133 A·cm^−2^·M^−1^ and detection limit as low as of 147.54 nM. The prepared sensor was quite selective and worked selectively in the presence of interfering ions. Multiphase solid materials with nano-dimensional ZnO particles have also been used for the development of effective sensors for hydrazine molecules. These multiphase solid materials with ZnO have a direct influence on their size, interfacial properties, and electrocatalytic activities against harmful analytes [58]. It was found that most of the electrochemical-based sensing of hydrazine molecules using ZnO nanoparticles have mainly used nafion for the tight attachment of ZnO nanomaterials on the surface of gold electrodes. These nafion molecules have the ability to form net-like layered structures on the surface of electrodes which form a partially blocked array system on the electrode surface [59]. These nafion molecules further coat the ZnO particles on the surface of bare gold electrode, and are helpful in the passivation of the electrode surface and further decrease the electroanalytical performance against external agents. In order to overcome this problem, researchers have recently used carbon nanotubes (CNTs) along with ZnO nanomaterials for the modification of the electrode surface in chemical sensing applications [60]. The presence of CNTs further affects the availability of accessible surface area for electrocatalytic activities. Moreover, the presence of CNTs further lowers the electrical resistance and enhances the mechanical strength and stiffness of the modified electrode during the analysis. The charge transport ability and chemical stability of the ZnO particles were further enhanced by using the CNTs.

### 3.2. Nitrophenol Chemical Sensor

4-nitrophenol (4-NP) is a distinguished type of aromatic organic pollutant, considered as a noxious and bio-obstinate complex which is harmful to various vital organs, including the central nervous system, liver, kidney, and blood of living organisms, and produces several diseases [61]. The higher stability and lower solubility in aqueous media makes it difficult to degrade to non-hazardous products. In this regard, the catalysis of this organic pollutant by employing the ZnO nanomaterials have been proven as an effective environmentally-friendly and economical means of detecting and removing these harmful analytes. For instance, Thirumalraj et al. [62] produced a very easy and responsive electrochemical means for the estimation of 4-nitrophenol (4-NP) in different types of water samples by the application of ZnO nanoparticles. The used particles were functionalized using chitosan (CHT) molecules, and the obtained nanoneedles of ZnO were coated on the surface of screen-printed carbon electrode for the estimation of 4-nitrophenol. The formed sensor has several advantages, such as high sensitivity, low working cost, and good reproducibility as compared to conventional methods. The working procedure for the developed sensor was quite easy and economical with very rapid response to the analyte. The presence of chitosan provided extraordinary properties to the ZnO nanostructures, including the presence of high surface area with complimentary electronic properties with higher biocompatibility with the surface of nanoparticles. The detection measurements for 4-nitrophenol were carried out in 0.05 M acetate buffer media at pH ~ 5 with scan rate of 50 mV/s. The comparative studies were also carried out with bare, ZnO nanoparticles-, and chitosan-functionalized nanoneedles at a scan rate of 50 mV/s. The modified electrode displayed a weak cathodic peak at −0.793 V which was associated with the reduction of 4-NP to hydroxylaminophenol. The conversion mainly involves a four electron and proton transfer electrochemical process. The corresponding reversible peaks were observed at 0.373 and 0.150 V. The surface modification with the chitosan provided the appearance of small sub-units on the surface of the electrodes which acted as electro-active centers for the transference of electrons and provided a better material for the electrocatalytic measurements of 4-nitrophenol. The nature of the reaction media (e.g., the chosen buffer and the pH of the reaction media) played a significant role in the detection ability of ZnO-modified electrode against nitrophenol. To optimize these parameters, three different types of electrolytic solutions (0.05 M PBS, 0.05 M citrate buffer, and 0.05 M acetate buffer) were chosen and optimized for the catalytic detection of nitrophenol molecules. Among the chosen electrolytic solutions, it was found that the CV response of nitrophenol was maximum for the 0.05 M acetate buffer solution as compared to the other buffers. Therefore, studies varying scan rate and concentration were performed in the presence of 0.05 M acetate buffer. The other crucial parameter (i.e., pH) was also optimized by varying it from 2 to 14. The outcomes have pointed to the presence of a strong peak at pH ~ 5. The peak strength was found to decrease by decreasing or increasing the pH of the reaction media. Therefore, the pH 5, 0.05 M acetate buffer is an optimized buffer solution for the electrochemical measurements of nitrophenol.

These ZnO-based nanostructures have the ability to act as an effective sensitizer for light-mediated redox reactions. The electronic structure of ZnO molecules was mainly associated with these light-induced redox processes. These ZnO nanostructures possess well-filled valence bands and an empty conduction band. When an external photon with threshold energy equivalent to or greater than the band energy of ZnO was applied to the particles, there was the probability of electron transference from the valence band to the conduction band in these nanostructures. This kind of transference has the ability to produce a hole in the valence band and an extra electron in the conduction band. Being unstable in nature, these excited conduction and valence bands have the ability to recombine these electrons and holes and liberate a respective amount of energy in the form of external heat and get ensnared in the respective metastable surface states in nanostructures. These surface states were further reacted with the electron donor and electron acceptor species and further adsorbed on the external surface of ZnO. The corresponding electrical double layer of the adsorbed species was formed on the surface of ZnO materials. These charged species have the ability to act as scavengers or surface defect mediators to catch the available electrons or holes in these nanostructures. Thus, the corresponding recombination of holes and electrons is averted and following redox reactions have the chance to occur on the ZnO surface. Undoped ZnO has n-type conductivity, which can be assigned to the asymmetric doping restraints and tendency towards defects or impurities. *p*-type doping with Ag, P, N, etc. has the tendency to modulate the conductivity and electrical and catalytic activities of ZnO nanoparticles. It has been well-established in the literature that the photocatalytic performance of ZnO was greatly improved by doping the surface of ZnO nanostructures with Ag. The electron transference rate was found be highly influenced in the presence of Ag [63]. For instance, Divband et al. [63] used the efficacy of Ag-doped ZnO nanostructures for the photocatalytic degradation of 4-nitrophenol (4-NP). The measurements were mainly done with the 50 mL solution of 10 ppm concentration of 4-nitrophenol. The prepared solution was thoroughly stirred with the help of magnetic stirring in the dark for at least 15 min. This stirring is helpful for the establishment of an effective adsorption/desorption equilibrium between the 4-nitrophenol on the external surface of ZnO. After equilibration in darkness, the respective solution was placed in ultraviolent light under stirring conditions. The samples were removed after a particular time frame and centrifuged at 1500 rpm to remove the catalyst particles. The respective measurements for the concentration of 4-nitrophenol were made by using UV-visible spectroscopic techniques. The absorption intensity appeared to be decrease at 315 nm with time. This behavioral aspect was related to the oxidative degradation of 4-nitrophenol in the presence of ZnO nanoparticles.

With the introduction of microreactor technology in photocatalysis, 1D nanomaterials of ZnO have been comprehensively investigated as a new method to amend the internal wall of capillary microchannels with nanostructures. Being a wide band gap material, ZnO has shown tremendous potential as an effective catalyst to degrade unrelenting organic pollutants from the environment. 1D ZnO micro-nanostructures have the ability to form an effective microchannel inside the internal wall of a capillary. The formed nanorod of ZnO has the tendency to uniformly arrange on the internal wall of capillary microchannels and provides a better composite catalyst or device for the degradation studies. Conversely, the one main disadvantage of these 1D nanorods and nanotubes of ZnO-based nanostructures with vertical heights of around 2–5 μm is the lesser tendency to make complete utilization of the full internal space inside capillaries of microchannels [64]. Therefore, by amending the properties of as-prepared ZnO-based nanostructures, there is a great deal of room for the improvement of the sensitivity of the as-prepared sensor. In this regard, Zhang et al. [64] prepared grass-like double-layer micro-nanostructures of ZnO in restricted microchannels by using a fluid construction process. The developed height of the grass-like structures was found to be 50 μm. The potential use of these grass-like catalysts was checked against the degradation of o-nitrophenol molecules. The outcome of the work revealed that capillary microreactors of ZnO have the tendency to solve the problem of the stability of the developed sensor, and the formed sensor was quite selective and sensitive against the chemical reduction of o-nitrophenol. The association of surface-connected processes with the quantum confinement effects in ZnO-based nanostructures resulted in admirable optical and electrical properties of nanostructures that can be adjusted to tackle particular requirements. In addition, the porous morphologies of these ZnO nanowires further assists the quick distribution of analyte species to binding locations, leading to a quicker reaction rate in the developed sensor. Additionally, the zinc oxide (ZnO) nanowires have the advantages of an easy and cost-effective growth process on the surface of insulating materials. These properties have further facilitated sensor fabrication, as the growth of ZnO nanowires can be simply managed to produce a conductive mesh-like arrangement on the external templates, thereby decreasing the production cost of sensors. Moreover, simplicity in fine-tuning the optical properties of ZnO nanostructures by using the defined nanowire diameters might afford added benefits in terms of a composite opto-electronic podium for the sensor development.

Gupta et al. [65] used heterostructures of a ZnO nanowire with surface modifications with pyrenebutyric acid (PBA) for the effective sensing of trace amounts of p-nitrophenol in biological systems. It was found that the fluorescence intensity of the prepared ZnO nanowires was significantly reduced in the presence of p-nitrophenol. Therefore, the detection was based on the degree of the fluorescence quenching in the presence of external analyte. The collision quenching of ZnO with the external moiety is commonly acknowledged as the central de-excitation path, where energy transmission from the π* orbital of pyrenebutyric acid to the π orbital of p-nitrophenol occurs. This process permits exceptionally high receptive recognition of analyte that could facilitate the detection of very minute amounts of p-nitrophenol in biological systems. These ZnO-based nanowires have offered a strong sustainable configuration for implanting the receptor molecules, while in parallel it is advantageous for the fabrication of sensory devices due to the economical processing method. Similarly, Singh et al. have employed composite structures of ZnO with CeO_2_ nanoparticles for the detection of p-nitrophenol in aqueous media [66]. The formed composite nanostructures had good control over the crystallinity and optical properties of ZnO nanoparticles, and acted as a good electron mediator for the detection of external analytes. The as-prepared chemical sensors displayed sensible, selective, and reproducible sensitivity of around 0.120 µA/(nM·cm^2^) with a detection limit of 1.163 µM. This kind of analysis unfolds the ways in which the simply synthesized CeO_2_–ZnO can competently be used for sensor fabrication. The nanoplates of ZnO nanostructures on the surface of ITO substrate also provided a better catalytic ability for the detection of 4-nitrophenol [67]. The analysis was mainly achieved in the absence of reducing agent. The catalysis was found to be influenced by the ultrasonication waves under room-temperature conditions. The respective conversion rate for 4-nitrophenol to 4-aminophenol was found to be as high as 4.483 × 10^−2^ mol·min^−1^. The presence of a high content of oxygen vacancies on the surface of (001) faceted nanoplate of ZnO was assumed to be the driving force for catalytic conversion of 4-nitrophenol to 4-aminophenol.

### 3.3. Nitroaniline Chemical Sensor

The derivatives of anilines such as nitroaniline are extensively employed in pharmaceutical, polymer, rubber, dyes, paints, and explosive industries. The surplus discharges of these pollutants in surroundings have destructive consequences on living beings and the environment. These chemical substances are highly lethal towards aquatic life, lead to adverse effects on the liver, and cause methemoglobinemia in humans. Additionally, ingestion and absorption of these toxins through the skin produces an allergic reaction. As a result, there is an increasing awareness among scientists of the necessity to develop an uncomplicated, time effective, highly stable, selective, and sensitive method for the estimation of this harmful chemical. In this area, ZnO has received more attention due to its unique and distinct optical and electrical properties. The availability of high crystallinity at low working temperature with modifiable electrical properties with good control over the biocompatibility of ZnO makes it a promising material for the detection of nitroaniline molecules with great accuracy and precision.

**Figure 4:**
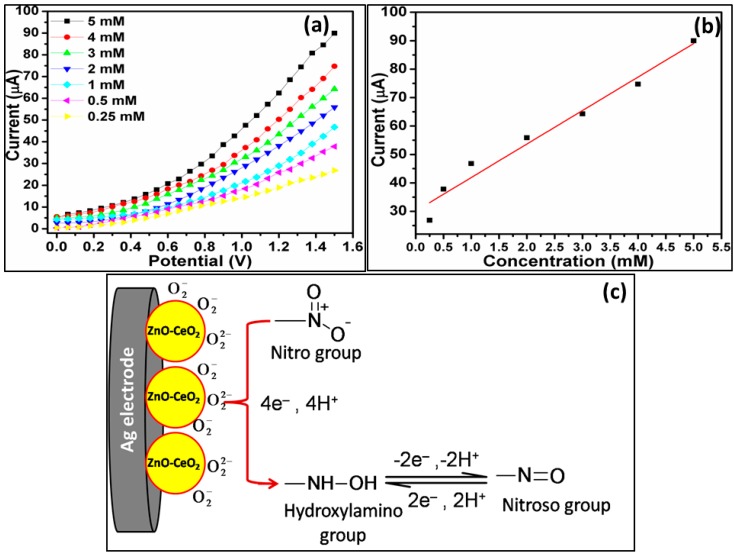
(**a**) Current-voltage responses for various concentrations of nitroaniline; and (**b**) Calibration curve for nitroaniline using ZnO-doped CeO_2_ nanoparticles-modified silver electrode (AgE); (**c**) A proposed sensing mechanism for the ZnO-doped CeO_2_ nanoparticles-modified AgE toward nitroaniline sensing. Adapted figure from [69] with permission from copyright (2016), Elsevier.

Recently, Ahmad et al. developed a binder-free, highly reactive, and sensitive chemical for the detection of p-nitroaniline by using ZnO nanoparticles [68]. Nanorods of ZnO were used for the modification of fluorine doped tin oxide (FTO) electrode. The main advantage of the method is that the analysis was performed at low-temperature conditions in aqueous medium. The developed sensor displayed incredibly high stability and reproducibility. Cyclic voltammetry (CV) peaks with nanorods of ZnO on the surface of FTO electrode in PBS with pH ~ 7.0 were observed in the potential range from 0.4 to +1.0 V. The scan rate for the measurements was kept at 100 mV/s. The reference electrode during the analysis was Ag/AgCl (saturated KCl) solution. The modified electrode in the absence of p-nitroaniline showed no current peak. The well-defined peak was observed in the presence of p-nitroaniline at +0.55 V. The obtained current peak was associated with the electrocatalytic oxidation of p-nitroaniline on the surface of modified FTO electrode with ZnO nanorods. The influence of the presence of external binder was also checked in the CV response for modified electrode in presence of p-nitroaniline. Figure 4b shows that in the absence of any kind of external binder, the electro-catalytic response against p-nitroaniline was found to be better as compared to the results in the presence of binders. The reactive ionic species which were converted from dissolved oxygen molecules in the reaction media in the presence of ZnO nanoparticles have the ability to absorb electrons from the conduction band of the particles. These reactive species were further reacted with the p-nitroaniline and oxidized it to respective CO_2_ and H_2_O molecules after transitory movements of numerous intermediary responses.

**Figure 5:**
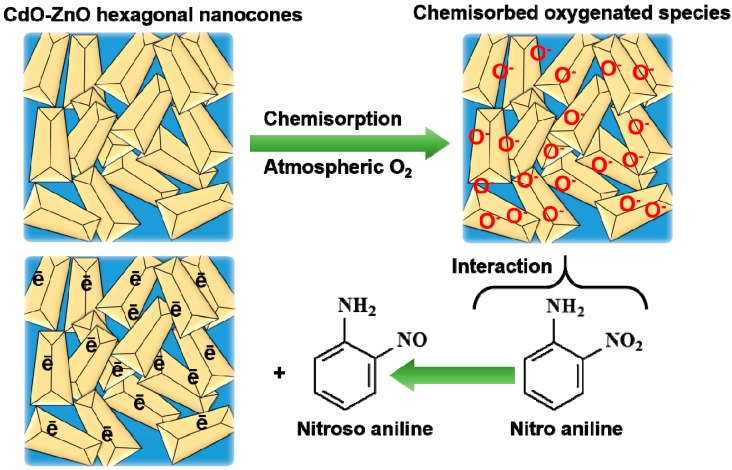
A sensing mechanism for nitroaniline sensing using modified GCE with CdO-ZnO hexagonal nanocones. Adapted figure from [70] with permission from copyright (2017), Elsevier.

The potential catalytic properties of ZnO nanostructures were further enhanced by doping the nanostructures with other lanthanide oxide materials. The doping of the nanostructures lends the ability to increase the reactive surface area of the particles, the number of defects, and the respective amount of oxygen vacancies on the particles. The effective diffusion rate for the electron transportation is also influenced by the presence of dopants. For instance, the combination of CeO_2_ with ZnO has the ability to form n–n type hetero-junctions for the electron transference reactions (Figure 4). The transference of electrons results in the formation of depletion around the interface of the nanocomposites, and improves the catalytic properties of the formed sensor [69]. A similar analysis was also performed with the help of the bi-composites of CdO-ZnO hexagonal nanocones for the detection of nitroaniline. The prepared sensor displayed a sensitivity of ~129.82 μA·mM^−1^ cm^−2^. The distinctive hexagonal nanocones of ZnO provided a high surface-to-volume ratio, which was found to be accountable for producing a huge amount of reactive oxygen groups on the surface of the CdO-ZnO nanocones and eased the oxidation process of nitroaniline (Figure 5) [70].

**Figure 6:**
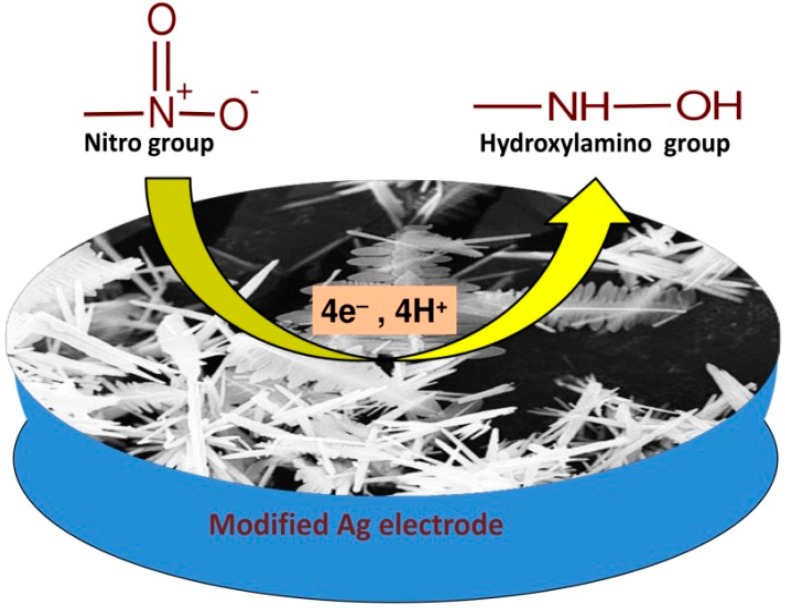
A schematic representation of the electrochemical sensing mechanism for the Sm_2_O_3_-doped ZnO beech fern hierarchical structures-modified AgE toward nitroaniline sensing. Adapted figure from [71] with permission from copyright (2017), Elsevier.

The role of Sm_2_O_3_-doping on the hierarchical structures of ZnO was also used for the generation of an electrochemical sensor for nitroaniline. The synthesis of the biocomposites was done by employing a simple, very economical, and fast hydrothermal method. The starting materials for the preparation (i.e., Zn(CH_3_COO)_2_·2H_2_O and Sm(NO_3_)30·6H_2_O) are easily available. The synthesis was mainly carried out at 155 °C for 7 h in basic media with pH of 9.5, respectively. The formed composite mainly displayed needle-shaped and leaf-shaped morphology, with particular outlines of ovate or triangular-ovate structures resembling ferns. The high surface area with good control over the electron transportation provided a better pathway for the electrochemical detection of nitroaniline. The sensitivity of the formed sensor was 1.71 μA·μM^−1^·cm^−2^ with detection limit of 15.6 μM [71]. Figure 6 exhibits the schematic representation of the proposed sensing mechanism for nitroaniline sensing using Sm_2_O_3_-doped ZnO beech fern hierarchical structures.

### 3.4. Ethanol, Methanol, and Propanol Chemical Sensor

The application of ZnO-based nanostructures for the recognition of venomous pollutants including inflammable complexes and organic compounds are an area of growing interest in both household and workplace atmospheres. For instance, Sahay and coworkers [72] investigated the application of thin films of ZnO nanostructures obtained by the spray method for the analysis of ethanol and acetone molecules. The corresponding effect of external dopants such as Fe, Al, In, Sn, and Cu on the response rate of a thin film-based ZnO sensor for ethanol was analyzed [73]. The sensing abilities of thin films of ZnO-based nanoparticulate were tested by Cheng et al. for the-sensing of ethanol and propyl alcohol molecules [74]. The developed sensors displayed higher sensitivity and selectivity for the tested molecules. The ability of thick films of ZnO-based nanostructures to simultaneously detect ethanol and propanol was achieved by Arshak and Gaiden. As for the sensing utilities of ZnO nanostructures, the presence of high Brunauer Emmett Teller (BET) surface area of the nanomaterial is a prime requirement for the analysis. In order to detect the optimum BET surface area for the preparation of sensor, a nanomaterial with highly porous morphology is necessary. In the current era of research, various ZnO morphologies, such as rods, belts, tubes, fibers, sheets, films, microflowers, and cones have been prepared with optimized porous character. For instance, the sensory reaction for nanoporous microbelts of ZnO was found to be very high for a diverse range of ethanol concentrations at a temperature of 300 °C. For a low concentration of around 1 ppm, the response rate was found to be 4.7. Upon increasing the amount of ethanol to 100 ppm in the aqueous media, there was an amazingly higher response value of 38.4. Moreover, the formed sensor showed a higher reversibility for ethanol [75].

These developed sensors work on the principle of variations in the values of conductance brought by the adsorption of an analyte on the exterior surface of the ZnO-based sensor. The atmospheric oxygen group has the ability to be adsorbed on the microporous structures of ZnO nanobelts. By the adsorption of electrons from the conduction band of nanomaterials [76], these oxygen molecules are converted into their oxidized form on the exterior surface of the sensor and form a substantially thick space-charge layer on the nanoparticles and amplify the potential barrier with an enhanced resistance during the analysis. These ethanol molecules have the tendency to respond against the adsorbed oxygen molecules and form CO_x_ and H_2_O molecules with the discharge of electrons at a reasonably high temperature of 300 °C. During the analysis, the decrease in the oxygen coverage results in the thinning of the depletion layer and the enhancement of conductivity in the presence of ethanol. On the basis of the resistivity of nanofilms of ZnO-based materials, the sensitivity of the formed sensor was varied drastically. The reaction temperature reversibly modulated the resistances of the nano films. The concentrations of external dopant on the surface of ZnO nanostructures further modified the resistivity of the sensor. It was verified that the higher concentration of dopant had a higher scattering efficiency, which in turn produced the enhancement of resistivity during the measurements. The use of three-dimensional (3D) nanoscale ZnO materials has received substantial consideration due to their astonishing features as applied in sensory devices. It is a well-established fact that the sensory aptitude of particles mainly depends on the microstructural properties of ZnO materials, which are affected by the method of synthesis, which latter affects the detection rate and sensitivity of the formed sensor. The growth behaviors of these three-dimensional (3D) nanoscale structures of ZnO are also affected by the variations in the external conditions of temperature, pH, and concentrations of the starting materials. The comparative studies have clearly shown that the sensing aptitude of ZnO nanoflowers was found to be much higher as compared to the nanowires, rods, or plates of ZnO materials. In addition, the sensor prepared by using these flowerlike structures of ZnO materials has shown considerably higher response of 24.1, 14.6, 14.2, and 13.8 for methanol, propanol, n-butanol, and acetone, respectively. The higher rate of diffusion of analytes in these 3D flowerlike ZnO nanostructure films is mainly responsible for the higher sensitivity. The resultant rate of adsorption and desorption was also found to be higher in these flowerlike nanostructures.

The use of the easy and consistent means of I–V techniques has shown the tremendous potential for the detection of methanol molecules with short response time. When methanol molecules were present in aqueous media, the obtained current response of ZnO nanoparticles was found to be changed significantly. This is mainly due to the adsorption of methanol molecules on the surface of ZnO nanoparticles [77]. The liquid phase analysis of the methanol molecules was mainly performed by using calcined samples of ZnO nanoparticles. The sensor was prepared by coating the surface of a glass electrode with a combination of a calcined ZnO-based structure and conducting agents. The formation of an even layer with high stability was achieved by drying the modified electrode in an electric oven at 60 °C. The respective I–V responses of for the liquid methanol were tested by ZnO thin-film as a function of current against potential. The corresponding time delay for the measuring device was 1.0 s. The obtained results clearly show that the current response of ZnO was increased with increase in the concentration of methanol at room temperature. These results have pointed out that the sensing mechanism for methanol molecules is a surface-based procedure. The obtained detection limit was tested by using a broad range of methanol concentrations. The obtained sensor displayed a methanol detection range varying between 0.25 mM to 1.8 M with a detection limit of 0.11 mM. The results clearly demonstrate that the exceptionally high surface area contributes greatly to the adsorption of external analyte and had a direct influence on the catalytic activity of the ZnO NPs for the estimation of methanol molecules. cn. ZnO molecules have the tendency to generate a constructive microenvironment on the exterior surface for the estimation of methanol molecules by the adsorption process. The enhanced sensitivity of ZnO-based nanostructures is attributable to the elevated rate of electron communication between the analyte and the used nanostructures. Higher stability with better reproducibility and shelf life were the additional benefits of the produced sensor. Methanol molecules have the ability to react with the adsorbed oxygen species on the exterior surface of nanostructures and are oxidized to formaldehyde and subsequently converted to formic acid by the release of electrons into the conduction band of the ZnO particles. As a result, there was a reduction in the resistance of the nonmaterial upon contact with the methanol molecules.

The thermodynamic studies for detecting the sensory mechanism for methanol and ethanol molecules have clearly demonstrated that the alcohols under investigation with identical molar masses have shown a reliance on packing arrangements. The more strongly-packed atoms on the exterior molecular structure of the nanoparticles displayed lower response time for the analyte being studied. This behavioral aspect can be elucidated by a rapid rate of adsorption on the exterior surface of semiconducting nanomaterials [78]. In addition, the boiling point of the adsorbed analytes also influenced the adsorption rate of the sensor. Molecules with higher boiling point had a greater tendency to adsorb onto the surface of the developed sensor. These phenomena were quite similar to the condensation process. Mainly, the adsorption was achieved by the breaking of respective O-H and C-H bonds in the alcohols, and the formed charged species were adsorbed onto the surface of the sensor. The adsorption process was well in accordance with the Freundlich isotherm equation. These isotherms facilitate the construction of the curves for the detection of sensitivity and the respective kinetics for the process. Being a well-liked drink, liquor has been consumed worldwide for thousands of years. With advancements in science and technology, there is a progressively greater diversity of liquors in the marketplace. In the interest of profit, the adulteration of liquor products is a known phenomenon, often with unsafe elements that harm the vital body organs. Therefore, means of differentiating the damaging components in liquors are catching the interest of scientists. It was found that these liquors are a complex combination of water and alcohol molecules as the major component, with hundreds of other small additional compounds. By detecting the harmful molecules in the liquor, we can easily judge the quality of the liquor. In this regard, a novel coplanar ZnO sensor was used for checking the purity of liquor. For the analysis, liquor was injected on the surface of a pure ZnO nanofilm which was previously screen-printed. The presence of external dopants such as transition metals was checked for the investigation of analytes [79]. For the sensor preparation, the bare gold electrode was first stamped on the dirt-free clean surface of an alumina ceramic chip by screen printing methodology. Then, the formed electrode was sintered at 850 °C for 15 min. The resistor paste RuO_2_ was printed on the modified electrode by screen printing. After that, the corresponding paste of ZnO was printed on the formed chip. The obtained area for the detection of analyte was about 0.5 mm × 1 mm, with a thickness of about 10 µm. The obtained sensor displayed a highly porous sponge-like thick film of ZnO for the detection of harmful pollutants. This porous nature of ZnO nanostructures provides different paths and enhanced surface area for the mass movement of target species. In addition, the hierarchically porous nanorod-like ZnO structures have displayed good response towards ethanol and acetone molecules. At low ethanol and acetone concentrations of around 1 ppm, the obtained responses were about 3.2 and 3.3, respectively, as compared to 24.3 and 31.6 at concentration of 100 ppm of ethanol and acetone in the reaction media. In order to study the repeatability of the sensor, the response analysis was performed by using the exposure to 100 ppm ethanol at 320 °C up to ten times. It was observed that the average value of the obtained sensitivities was 24.5, with the changeability of the sensitivity being less than 2.1%. All of these outcomes clearly show the good repeatability of the ZnO-based sensors. The operating temperature also influenced the working potential of the ZnO-based sensor for ethanol and methanol molecules. At an operating temperature of 200 °C for diverse range of concentrations of ethanol and methanol, the developed sensor did not show any considerable response against methanol. This was mainly explained by the presence of insufficient thermal energy in the system to respond with the adsorbed oxygen species on the exterior surface of the nanoparticles. The presence of a space-charge layer on the surface of nanoparticles has the tendency to hinder the charge transportation at low-temperature conditions. On the other hand, as the working temperature was enhanced during the measurements, the response rate of the ZnO-based film to methanol was augmented for all studied concentrations. The electron transport and the respective surface reactions progressed quickly with the enhancement of thermal energy. Among the various temperature ranges evaluated, the response rate was maximum in the region of 275–300 °C. This is mainly explained by the presence of an adequate amount of adsorbed oxygen species on the surface of the ZnO film, which respond most efficiently to the methanol molecules. On the other hand, for the ethanol molecules, the enhancement of the response rate with the operating temperature was comparatively less for concentrations up to 200 ppm. Upon further increasing the concentration to 250 ppm, the response rate was found to be enhanced up to 250 °C and then started to decline.

This is mainly explained by the enhancement of surface coverage by ethanol molecules on the surface of ZnO films, which stopped the successive adsorption of atmospheric oxygen on the surface of the films, causing the chemical reaction to progress with a slow rate, and thus a decline in response occurred.

In addition, the pH of reaction media also played a significant role in the sensing ability of nanostructures. The pH of the reaction media is considered as one of the primary components and is an easily computable and amendable factor during the fabrication of nanostructures that influences the structural and morphological behavior of particles. Studies have shown that the variation of pH values during the synthesis of particles affected the shape and morphology of the generated structures. By simply varying the pH, one is able to achieve particle morphologies ranging from plate-like to flower-like. The surface area of the particles is also affected by varying the pH of the reaction media, and hence the sensing ability was influenced by varying the reaction conditions. In the case of nanorods of ZnO-based particles, it was observed that at pH ~ 8, the formation of nanorods was well-defined. This was mainly explained by the minimization of the total energy of the system by the unstructured and spontaneous polarization of the reaction system. This polarization further affected the non-centrosymmetric crystal structures of the obtained nanorods of ZnO in the reaction media. On further enhancing the pH of the reaction media to 10, there was a tendency of these formed nanorods to agglomerate. This may be explained by taking the effect of reaction solution super-saturation with enhancement of the system pH. The nanorods then lost their shape to form joined agglomerates, minimizing the overall energy of the reaction system. The obtained particles displayed a higher sensitivity for methanol, ethanol, and propanol at different reaction temperatures [80]. On interpreting the results, it was found that the response rate of the prepared sensor varied as the pH of the reaction media varied. The particles prepared at pH 11 had a greater response than particles prepared at pH ~8. The explanation for this augmentation in the sensitivity at higher pH values may be attributed to the morphologies of the formed particles.

**Figure 7:**
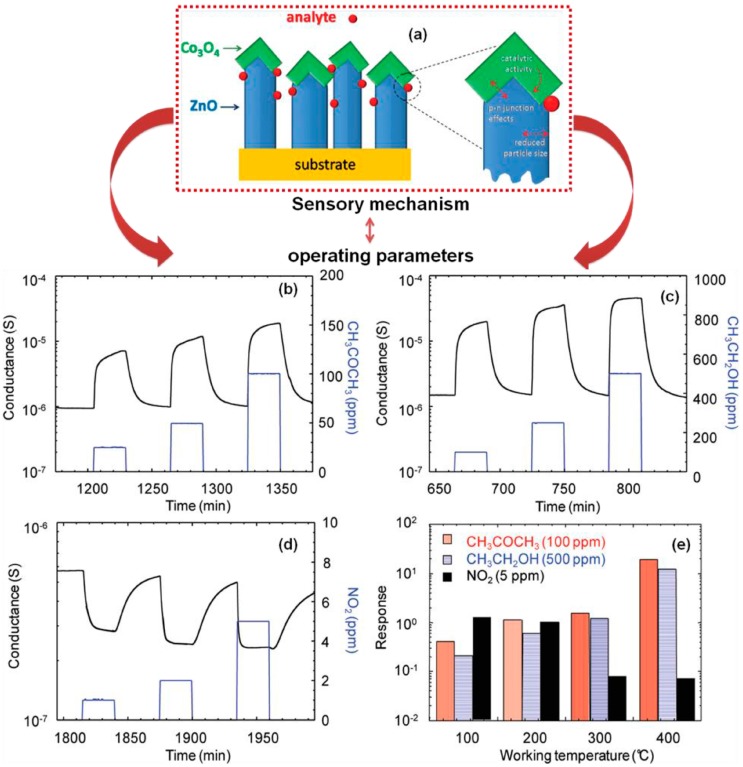
(**a**) Schematic representation of the main phenomena beneficially affecting the sensing behavior of the present Co_3_O_4_/ZnO nanocomposites. Sensing responses (black) of a Co_3_O_4_/ZnO sensor (specimen ZnCo_10_) toward square concentration pulses (blue) of (**b**) CH_3_COCH_3_; (**c**) CH_3_CH_2_OH; and (**d**) NO_2_. Working temperatures were (a) 400 °C and (b) 200 °C; (**e**) Dependence of the response on the operating temperature for selected analyte concentrations (specimen ZnCo_10_). Adapted figure from [81] with permission from copyright (2012), American Chemical Society (Washington, DC, USA).

The bi-composites of p-Co_3_O_4_/n-ZnO particles were also used for the fabrication of an effective sensor for ethanol molecules. The advantage of this method is that it has also been applied for the detection of acetone and nitrogen dioxide molecules with great accuracy and efficiency. For the synthesis of biocomposites, the ⟨001⟩ oriented nanorods of ZnO nanoparticles were first raised on the surface of alumina substrates by using the plasma-enhanced chemical vapor deposition (PECVD) technique. The as-produced templates were further employed for the growth of nanograins of Co_3_O_4_ molecules. To use the prepared particles for practical sensing applications, the selectivity of the formed sensor was optimized as a function of time, concentration, and temperature. Out of all the studied parameters, the working temperature was established as one of the crucial factors for detecting the harmful analyte. The sensing ability of the developed sensor was found to be superior to those of earlier representative examples of Au-doped or F-doped Co_3_O_4_ nanoparticles. Mainly, the p-Co_3_O_4_/n-ZnO junctions have the ability to generate a better charge partition on the interface of the two used oxides (Figure 7). The formed junctions further affected the conductance of the system and modulated the interaction of the target species with the nanoparticles [81].

**Figure 8:**
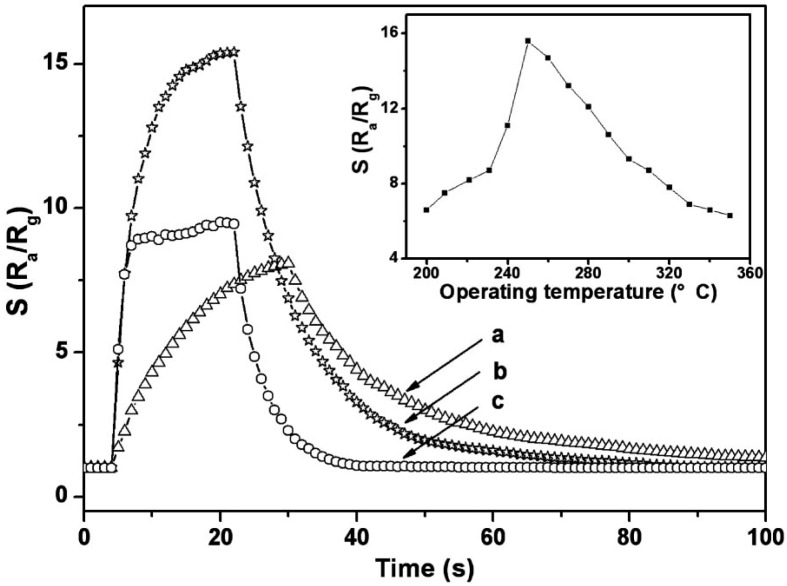
Response of the present ZnO to 10 ppm ethanol at various operating temperatures of (**a**) 220; (**b**) 250; and (**c**) 300 °C. The inset shows the sensitivity to 10 ppm ethanol at operating temperatures in the range of 200–350 °C. Adapted figure from [83] with permission from copyright (2009), American Chemical Society (Washington, DC, USA).

It was also observed that hierarchical microspheres of ZnO particles demonstrated higher potential for carrying out electron transportation and response against external analytes. The higher rate of kinetics was mainly due to the presence of high surface areas in these hierarchical structures. The analyte molecules had a higher rate of diffusion on the surface of the particles and enhanced the sensitivity of the developed sensor. The applications of these hierarchical microspheres of ZnO particles with the cataluminescence responses against ethanol molecules have shown tremendous importance in the measurements. The prepared sensor showed a lesser consumption of energy and superior response, with high sensitivity and selectivity for ethanol molecules. The developed sensor could be one of the potential ways to solve the problem of detection in lower concentration ranges. The flow rate of analyte also influenced the sensitivity of the formed sensor. It was found that in the presence of a high concentration of ethanol molecules, the flow rate was high and the subsequent catalytic reaction was greater, enhancing the signal intensity during the measurement. The sensing mechanism was explained by two connected steps: one of the processes involves the formation of excited species during the analysis, followed by their relaxation after losing radiation. The other process comprised the recombination of charge carriers initiated from the external surface states of the ZnO nanostructures. The chemically adsorbed species on the external surface of the particles had the tendency to be converted into reactive centers, and enhanced the catalytic rate of the reaction. On the other hand, nanowires of ZnO particles had a higher response rate against ethanol molecules. The fabrication of the nanowires was easily achieved by the chemical vapor deposition method. The microelectromechanical system (MEMS) technology also affected the sensory aptitude of the ZnO particles. The sensor displayed high sensitivity with rapid response towards ethanol molecules at a working temperature of 300 °C. It was also seen that at very low concentration of 1 ppm of ethanol and n-butanol, the sensitivity was around 2.2 and 3.4, respectively. At a very high concentration of 100 ppm of these analytes, the virtual responses of the formed sensor were found to be 22.6 and 18.2, respectively. The formed sensor was found to be quite stable, with higher reproducibility. At the same working temperature, nanoflowers of ZnO displayed higher sensitivity than the ZnO nanowires. The primary reason for this variation was the difference in the surface area of the formed particles and differences in the reactive centers on the exterior surface of particles. The recovery time for the prepared sensors was 5 s and 18 s, respectively, for nanowires and nanoflowers of ZnO [82]. On the other hand, if porosity was introduced in ZnO nanowires, the respective response rate was significantly enhanced in the presence of ethanol or methanol molecules. The introduction of porous nature in these nanowires was mainly done by calcining the particles at a known temperature. This kind of heat treatment has the ability to decompose the used starting materials into wire-like zinc hydroxide carbonate molecules. The BET analysis showed the development of 39.1 m^2^·g^−1^ of surface area of ZnO nanowires. These porous structures showed a higher rate of diffusion of molecules with a higher rate of mass transportation in the sensing material. The sensitivity and the response time were further improved by varying the reaction conditions such as pH, temperature, and analyte concentration. The development of an ethanol sensor by using flowerlike bundles of ZnO nanorods was done by Zeng et al. [83]. The formed sensor showed a higher sensitivity of around 154.3 and superior reversible response rate towards ethanol for concentration ranges varying from 1 ppm to 100 ppm. The sensing mechanism was checked at various operational temperature ranges as a function of ethanol concentration. Figure 8 shows the response curves for ZnO particles in the presence of 10 ppm of ethanol molecules for different temperatures (220, 250, and 300 °C). From the data, it is seen that the sensitivity of the sensor had a maximum of about 15.6 at the working temperature of 250 °C. Upon increasing the temperature of the system, the response and recovery rate of the developed sensor was found to decrease. This can be explained by considering the kinetics and mechanism of ethanol adsorption on the surface of ZnO particles. Thus, it is clearly verified that the nanorods of ZnO particles have the potential to act as an effective chemical sensor against ethanol molecules with the use of low power. The higher response rate of the developed sensor at low operating temperature has further enhanced the scope of ZnO particles in rapid sensing applications.

**Figure 9:**
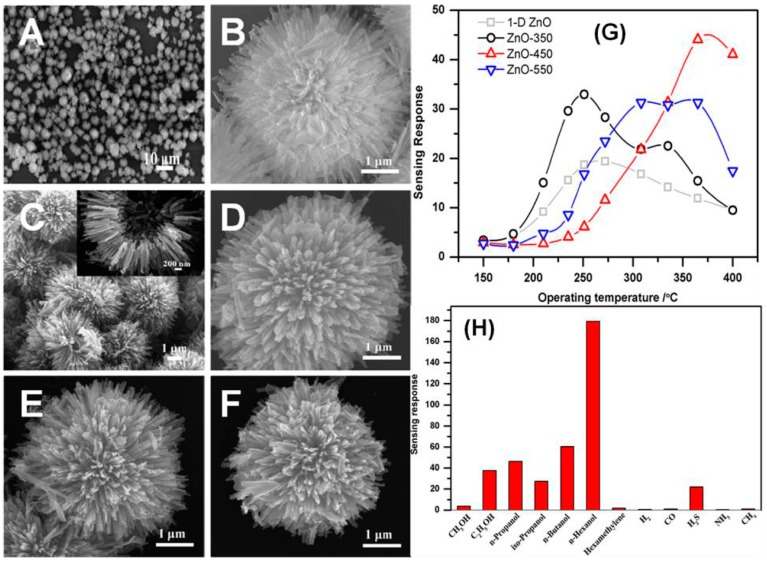

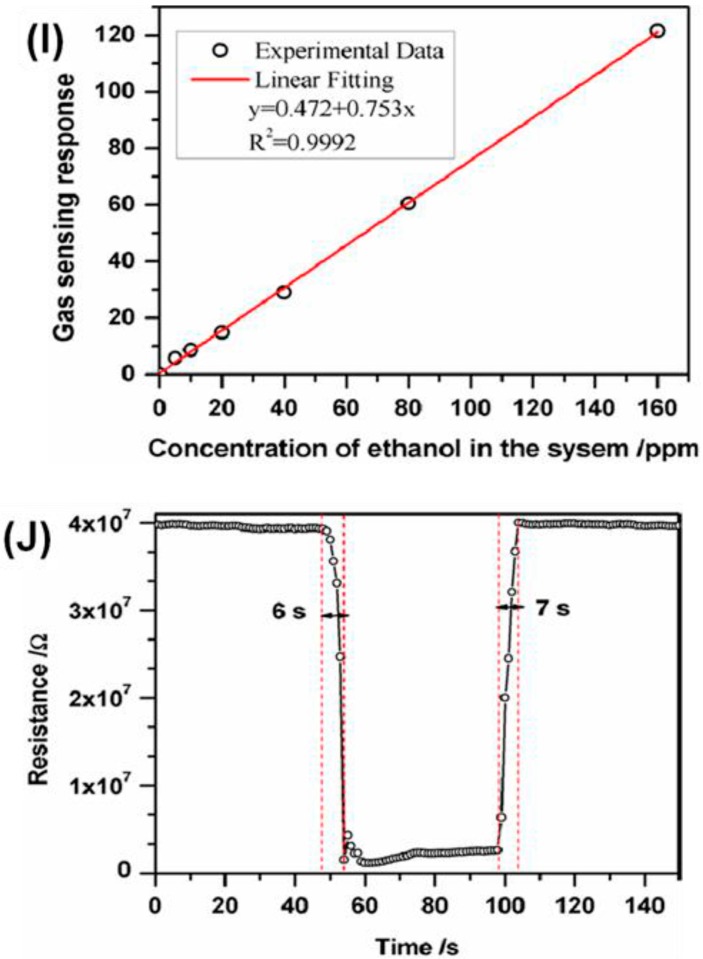
SEM images of the samples: precursor (**A**,**B**); ZnO-350 (**C**,**D**); ZnO-450 (**E**); and ZnO-550 (**F**), the inset in C presents a hollow structure of the ZnO-350; (**G**) ZnO sensors response to 50 ppm ethanol under different operating temperature; (**H**) Response of ZnO-350 dandelion-like hierarchitectures toward 50 ppm of different interfering molecules at the optimum operating temperature of 250 °C; (**I**) Response curve and linear fitting curve of the sensing response of ZnO-350 to different concentrations of ethanol at the operating temperature of 250 °C; (**J**) response and recovery time of ZnO-350 to 50 ppm ethanol at the operating temperature of 250 °C. Adapted figure from [84] with permission from copyright (2014), American Chemical Society, (Washington, DC, USA).

Fan and co-workers synthesized dandelion-like hollow ZnO particle hierarchitectures, and used them for the sensing of ethanol molecules. In total, three different structure types were prepared by heating the precursors at 350, 450, and 550 °C in the presence of air for approximately 2 h. The heating was controlled with a heating rate of 5 °C·min^−1^ during the synthesis of particles termed as ZnO-350, ZnO-450, and ZnO-550, respectively (Figure 9A–F). In order to compare the results, the flower-shaped ZnO particles were also prepared by hydrothermal methods, and their sensing ability was tested against dandelion-like hollow hierarchitectures of ZnO. The development of the sensor was done by dispersing the formed particles in the presence of ethanol molecules to form a homogenous colloidal solution. The as-formed dispersing solution was then encrusted on a ceramic tube having a pair of previously casted electrodes. The heating was performed by using a Ni–Cr alloy. The developed sensor was further aged at 200 °C for around 7 days before the analysis. Figure 9G illustrates the response of the ZnO particles used for the sensing of 50 ppm of ethanol molecules as a function of temperature. Form the data, it was observed that the response showed irregular behavior. First, the response rate increased, followed by a decrease in the response activity from 150 to 400 °C. It is clear that the detection of ethanol molecules was achieved by the adsorption and desorption of molecules on the available surface of ZnO particles.

**Figure 10:**
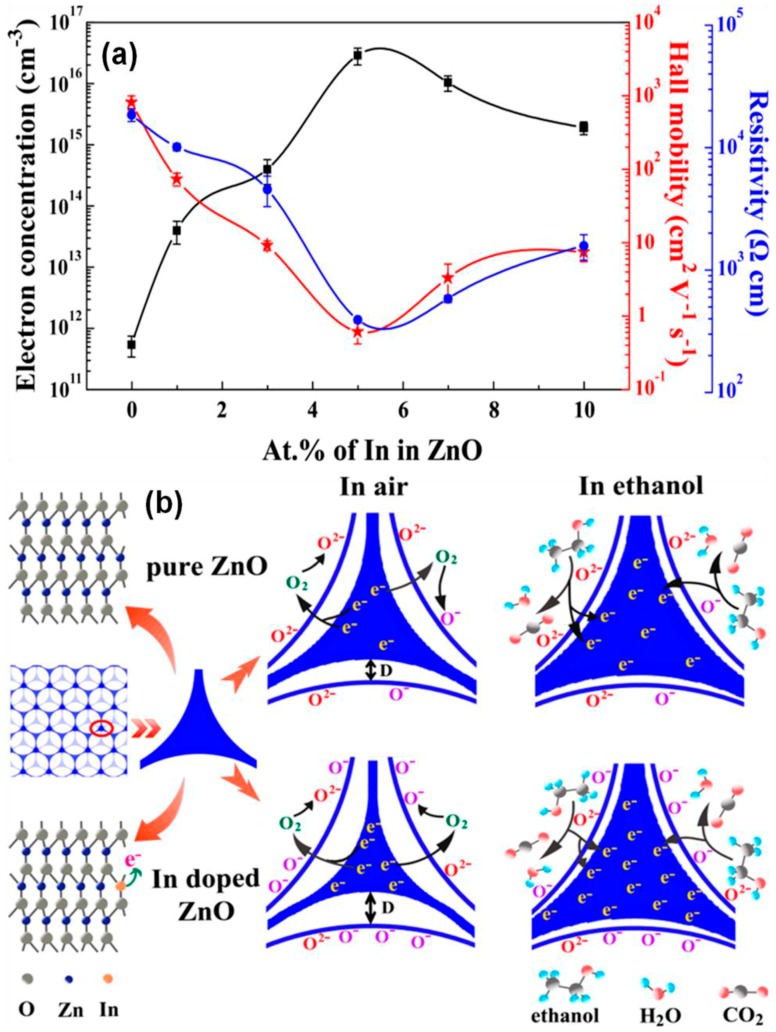
(**a**) Electron carrier concentration, resistivity, and Hall mobility of the 3 dimentional macroporous structures; the error bars represent the SD of the determinations for three independent samples; (**b**) Schematic diagram of ethanol sensing on the surface of pure and In-doped 3DOM ZnO. Adapted figure from [85] with permission from copyright (2016), American Chemical Society, (Washington, DC, USA).

An adequate amount of thermal energy provided by external heating is crucial to conquering the activation energy barrier for the chemically adsorbed molecules of ethanol on the surface of ZnO. The quantity of chemically adsorbed molecules of ethanol was enhanced with increases in the working temperature of the reaction media. On further enhancing the temperature, the process of desorption was the main factor decreasing the response behavior of the developed sensor. The optimum working temperature for the sensor was 250, 370, and 330 °C for ZnO-350, whereas the 1-D ZnO-based nanorods had the working temperature value of 450, respectively [85]. Moreover, the sensing response of the used ZnO particles was found to be highest for ethanol molecules as compared to other interfering molecules (Figure 9H). Figure 9I,J shows the variations of response as a function of ethanol concentration varying from 5 to 160 ppm for ZnO-350 at the temperature of 250 °C. In addition, the three-dimensionally highly-ordered macroporous nanostructure of ZnO particles also showed advantages for the detection of ethanol molecules. These macroporous structures had the ability to provide good control over the surface area of particles with high electron transference rate for these molecules, which enhanced the scope of particles for developing effective sensors. The external doping of ZnO particles with indium (In) is one way to improve the sensing properties of ZnO particles. The composites were mainly prepared by using the colloidal crystal templating method (Figure 10). While varying the percentage doping rate, it was found that 5% doping of in provided the highest sensitivity of ∼88% for 100 ppm ethanol molecules at 250 °C.

In comparison with the bare macroporous nanostructure of ZnO, the sensitivity was found to be three times higher. The colossal enhancement of the sensitivity to ethanol was associated with the amplification in the surface area and the electron carrier concentration in the prepared particles. The introduction of in provided additional electrons in the medium, which is supportive for escalating the quantity of adsorbed oxygen, guiding the high sensitivity of the sensor. The tetrapodal-like morphology with the presence of leg-to-leg linking of ZnO nanostructures was also revealed to be important in the investigation of very low concentrations of ethanol [86]. The electrical properties of ZnO nanoparticles were mainly used for the sensing of ethanol molecules. It was found that the use of UV light was quite effective for the excitation of electrons from the conduction band. Therefore, the use of external heating was avoided during the measurements. The respective plots of resistance in the presence of air and ethanol molecules were compared under UV illumination at room temperature. The excitation of electrons from the conduction band was generated due to the increase of photoelectrons by using the external photon absorption from UV light illumination. After switching off the UV illumination, the resistance value for the developed sensor returned to its usual value at the starting point. Conversely, in the presence of ethanol, the resistance values for the ITN-ZnO sensor were found to be astonishingly amplified, which is different from the case at elevated functioning temperature values. This enhancement of resistance was mainly associated with the *p*-type behavior of ZnO particles. The enhancement of resistance was also noted for different concentration ranges from 10 to 1000 ppm. The outcomes of the work have clearly illustrated the importance of the ability of a sensor to act at room temperature conditions for a wide range of concentrations.

### 3.5. Hydroquinone Chemical Sensor

Out of various types of phenolic molecules, p-hydroquinone (HQ) is considered as an essential compound in various types of biological and industrial practices involving the manufacture of coal-tar molecules, paper textile dyes, makeup products, and graphic developers. The excess breathing of HQ chemical has adverse effects on various vital organs, such as lungs, liver, kidney, and genito-urinary tract of human beings. In addition, the degradation of HQ is not simple in the current environmental circumstances. In this regard, ZnO nanostructures have received tremendous interest for the fabrication of effective sensors for HQ. For instance, Ameen and coworkers [87] used ZnO nanowhiskers for the detection of HQ by using the I–V technique. For the development of the sensor, a bare glassy carbon electrode was modified by using the ZnO nanowhiskers. It was observed that the presence of 10 mM of HQ in the reaction media considerably augmented the current value. The saturation current value was obtained after the concentration of 200 µM of HQ. The saturation is mainly explained to be due to the absence of free active sites on the surface of ZnO particles for the effective adsorption of HQ molecules. The developed sensor displayed a sensitivity of 99.2 µA·µM^−1^·cm^−2^ and a limit of detection of 4.5 µM, with a correlation coefficient of 0.98144 and response time of less than 10 s. The available active oxygen species on the surface of nanoparticles have the ability to react with HQ molecules, and oxidized HQ to 1,4-benzoquinone in the electrochemical analysis. The presence of the distinctive whiskers morphology of ZnO is useful for the production of an effective electrode material for the quick recognition of HQ.

In addition, the high electrocatalytic activity of ZnO-based nanoparticles for the electrochemical oxidation of hydroquinone was expressed by Freire et al. [88]. The sensing aptitude was tested by attaching the ZnO nanowires to carboxylic acid-functionalized multi-walled carbon nanotubes (MWCNTs). Comparison of the catalytic activity based on the preparation of ZnO particles was done by using the three different conditions involving the temperature variation in a hydrothermal microwave-based method. These variations in the synthetic conditions had the tendency to generate spherical, flower-like, and non-structured ZnO particles. From the CV analysis, it was found that due to the presence of high surface area, the flower-like morphologies of ZnO had better sensitivity towards HQ molecules. The lowest value of response rate was observed in irregularly-shaped ZnO particles. This was explained to be due to the inferior electron transport kinetics in contrast with the other composites of ZnO. In addition, these ZnO nanoflowers possessed a porous nature, which further augmented the surface area and supplied a minor mass transport obstruction and produced a rapid diffusion of HQ compounds from electrolytic solution to the modified electrode surface and enhanced the current intensity at this modified electrode. Recently, Ahmed et al. investigated the application of ternary metal oxides of ZnO with SrO and NiO (TMO) for the preparation of an HQ sensor [89]. The detailed spectroscopic studies have shown that the as-formed TMO displayed very unusual electronic and catalytic features compared to the individual oxides used for the preparation of mixed oxides. The formed TMO particles on the surface of glassy carbon electrode displayed the finest reactivity at a neutral pH range of 7.0. The mechanism mainly involved the transference of a coupled two-electron two-proton reversible reaction for the estimation of HQ molecules. Due to the presence of an adequate amount of active sites on the surface of TMO, molecules could provide a supporting nano-environment during the measurement of HQ molecules. The sensitivity, limit of detection, response rate, reproducibility, and stability of the developed sensor were found to be quite high for HQ molecules. Hollow and highly porous nanospheres of ZnO prepared by using the hard template process were used to enclose inside the nanosheets of graphene oxide for the development of effective engineered material for the detection of HQ molecules [90]. The effective electrostatic interactions between the ZnO particles and graphene oxide (GO) nanosheets were achieved due to the amino-functionalization of the particles. The presence of the highly porous structure of functionalized ZnO with tetra phenyl hydroxyl sulphate (TPHS) and GO sheets provided a large surface area for the adsorption of ethanol molecules. The electron transfer activity of the developed material was also found to be quite high for the estimation of analyte. For the HQ molecules, the respective peak was observed at 0.43 V, associated with the oxidation peak for HQ molecules, and the respective reduction peak was observed at −0.033 V on the surface of bare GCE. The engineered ZnO particles with GO particles on the surface of a GCE electrode displayed redox peak currents with 2.47-fold and 3.99-fold higher peak current values for the oxidation of HQ molecules. In addition, the peak-to-peak separation value was found to be 0.37 V, which was less than that of bare GCE.

**Figure 11:**
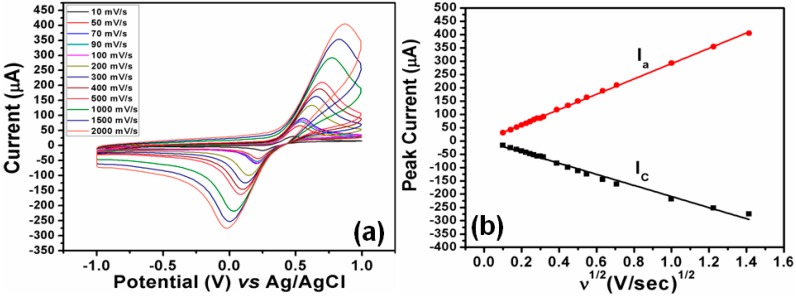

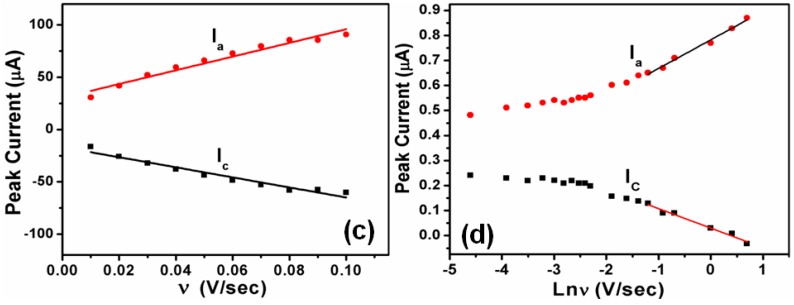
(**a**) Cyclic voltammograms obtained for ZnO nanoparticles/GC electrode in 0.1 M PBS (pH = 7.4), containing 5 mM hydroquinone at various scan rates of 10, 50, 70, 90, 100, 200, 300, 400, 500, 1000, 1500, and 2000 mV/s; (**b**) Plot for the anodic and cathodic peak current versus the square root of the scan rates in the same solution; (**c**) Plot for the anodic and cathodic peak current versus the scan rates in same solution; and (**d**) Plot for the anodic and cathodic peak current versus the natural Log of scan rates in the same solution. Adapted figure from [91] with permission from copyright (2014), American Scientific Publishers (Los Angeles, CA, USA).

On the other hand, the combination of ZnO particles as working electrode and Ag/AgCl with saturated KCl solution as reference electrode was employed for the estimation of hydroquinone [91]. The formed sensor displayed anodic (*E*pa) and cathodic (*E*pc) peak potential values at 0.431 V and 0.245 V for the 0.78 mM amount of hydroquinone. The obtained ratio of anodic (*I*a) and cathodic (*I*c) peaks was found to be 2.2. The ratio clearly points out the signals for the quasi-reversible process during the estimation of hydroquinone molecules. The prepared sensor also displayed higher conductivity, as the peak potential difference for the anodic and cathodic peak was found to be quite low. The effect of different scan rates has been visualized in Figure 11 for the developed sensor in the presence of 5 mM hydroquinone, showing its high sensitivity.

### 3.6. Acetone Chemical Sensor

**Figure 12:**
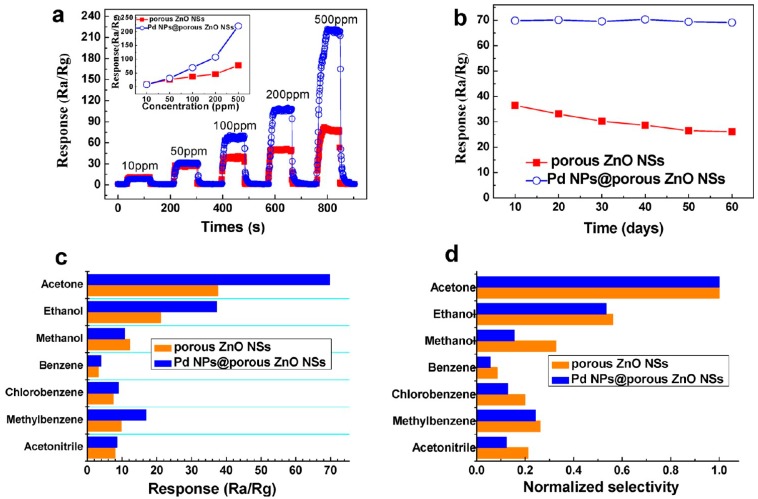
(**a**) Dynamic responses to acetone at concentrations ranging from 10 to 500 ppm of acetone sensors made with Pd−ZnO−Nnanosphere and ZnO−nanosphere at optimized operating temperatures. The inset shows response vs concentration curves of the corresponding sensors; (**b**) Stability studies of sensors exposed to 100 ppm acetone; (**c**) The selectivity of the acetone sensors to reducing gases; and (**d**) The corresponding normalized selectivity of sensors from (c). Adapted figure from [92] with permission from copyright (2012), American Chemical Society (Washington, DC, USA).

Among the various types of organic solvents, acetone is considered to be an effectual biomarker for the non-persistent identification of type-I diabetes in human beings, since this type of ailment has a direct effect on the concentration of acetone in the breath of human beings. It was found that the concentration of acetone was less than 900 ppb in healthy human beings, whereas the concentration rose to 1.8 ppm for type-I diabetes-affected persons. Therefore, preparing an effective method for the detection of acetone at very low concentrations is in high demand. In this regard, the effective electrical and conductive nature of ZnO makes it an efficient material for the preparation of chemical sensors for acetone. In addition, its diverse range of physical properties including electro-optical, acoustic, piezoelectric, wide band gap, and optimized luminescence properties make ZnO a promising material for the preparation of chemical sensors for acetone molecules. For instance, Xiao et al. [92] have used highly-porous and single-crystalline nanosheets of ZnO particles for the preparation of sensors. The synthesis of nanosheets was achieved by employing the annealing of hydrozincite (i.e., Zn_5_(CO_3_)_2_(OH)_6_) nanoplates. The solvothermal process was employed in a mixture of water/ethylene glycol as the solvent media. The effect of Pd coating on the surface of ZnO nanosheets was also evaluated to compare the efficacy of the prepared sensor. The doping of the Pd particles were mainly achieved by using the self-assembly method. In order to test the chemical sensing, the effects of the ZnO nanosheets were explored cautiously before and after the surface alterations with Pd nanoparticles. From the data, it was found that the chemical activity of the porous ZnO nanosheets was mainly due to the superior selectivity of the formed sensor with high response kinetics. This was mainly due to the higher contact of 2D nano-crystals of ZnO via the presence of (100) facets. The formation of the sensor was mainly done by dispersing the ZnO materials on the surface of adhesive Terpineol. The dispersion was prepared by the gentle grinding of particles in an agate mortar to form a slurry of the used materials. Then, this prepared suspension was cast on the exterior surface of a tube made up of ceramic material. The used ceramic tube also possessed two electrodes made up of Pt. As-coated material was dried at 80 °C. Subsequently, the dried tube was subjected to calcinations at 500 °C for around 1 h to generate thick films on the surfaces of the ceramic tube. The importance of the method was that no external conductive binder was mixed with the used material for the preparation of the sensor. The heating was done by using a small coil made up of Ni–Cr alloy. In order to attain stability, the prepared sensor was aged at 300 °C for one week. Figure 12 shows the responses to acetone as a function of temperature for the formed sensors with ZnO and Pd@ZnO, respectively. Upon looking at the plots, it can be seen that the response rate of the sensor to acetone molecules varied noticeably in the temperature range between 200 to 500 °C. The sensor made up of nanosheets of ZnO particles displayed the highest response of 37.5 to 100 ppm of acetone at 420 °C, and the responses declined further on enhancing the temperature. Modifying the surface of the ZnO particles with 0.5 wt % Pd nanoparticles led to a significant enhancement of 70 times as compared to ZnO nanosheets at the working temperature of 340 °C. Figure 12 displays the dynamic response and recovery of chemical sensors for acetone molecules prepared using nanosheets of ZnO and Pd-doped nanosheets of ZnO, respectively. From the data, one can easily visualize that Pd@ZnO-based sensors showed superior responses to the different concentrations The sensors prepared with Pd doping on the ZnO nanosheets had the tendency to achieve a reaction of ~222 to acetone molecules with concentrations as low as 500 ppm. The response for doped particles was found to be around three times higher than that of nanosheets of ZnO without any doping. The response rate and recuperation rate for Pd@ZnO sensors for 100 ppm acetone was around 9 and 6 s, respectively, compared to 10 and 7 s for nanosheets of ZnO-based sensors. The highly porous nature of Pd@ZnO sheets have a better ability to assist in the diffusion of acetone molecules, and enhanced the mass transport during the analysis.

The sensing mechanism for the acetone molecules on the surface of ZnO particles was explained as follows. It was observed that the atmospheric oxygen molecules were primarily adsorbed on the surface of particles as the heating of the prepared nanoparticles was performed during the initial phase [93]. On the other hand, the surface reaction was found to proceed further upon decreasing the working temperature of the media with a low speed. The electrons in the conduction band of nanoparticles were responsible for the generation of reactive species of oxygen on the exterior surface of nanoparticles. The desorption of these reactive oxygen species was done at 80, 130, and 500 °C, respectively. Of the various types of reactive species of oxygen, it was found that the O^−^ species were very stable and had the tendency to react with the molecules of acetone. The sensitivity of the prepared sensor was linearly dependent on the concentration of acetone and the working temperature. In order to investigate the influence of morphology, Zhang et al. [94] used hexagonal prism-shaped nanorods of ZnO particles with planar and pyramidal tips for the sensing of acetone molecules. The fabrication of these morphologies was mainly achieved using the Teflon-lined stainless steel autoclave method. The temperature was maintained at 60 °C for around 12 h. After obtaining the precipitation, the solution was subjected to centrifugation and subsequently calcined at 550 °C for around 2 h. The addition of Cr was also done to enhance the sensitivity and response behavior of ZnO nanoparticles for acetone molecules. The crystalline size of the formed particles were around 36.97 and 39.47 for pure and Cr@ZnO nanostructures, respectively. It was found that after the addition of Cr ion as dopant, the non-uniform rods of ZnO particles were converted into hexagonal structures with planar and pyramidal tips. The hexagonal wurtzite structure of ZnO nanoparticles possessed a basal plane with (0001) plane that terminated around the lattice point of Zn metal. The other basal plane has possessed oxygen lattice point in the pyramidal structures. The sensing for acetone was found to be quite low at room temperature conditions. Therefore, external heating of the samples was required for the detection of molecules. The response behavior was also affected by the concentration of acetone molecules. The Cr doping reduced the working temperature for the sensor and acted effectively for the analysis of low concentrations of analyte. Recently, the application of bi-composites of NiO with ZnO nanoparticles has been used for the detection of ppb concentrations of acetone molecules in reaction media [95]. The formation of the bi-composite occurred by the decoration of the exterior surface of ZnO nanomaterials with NiO particles by using the solvothermal technique. The hollow structures of ZnO particles have the ability to provide large surface area for the adsorption of analyte molecules. Primarily, the sensing ability was tested by optimizing the working temperature ranges for ZnO and NiO@ZnO nanospheres. These temperature-based characteristics of as-prepared sensors were calculated at a broad range of temperature in order to search the connection among the effective temperature and the acetone response. It was found that the response of the prepared sensors to 100 ppm acetone solution followed a volcano-shaped profile relationship between acetone response and working temperature for ZnO and NiO@ZnO nanospheres-based sensors. In terms of response rate, NiO@ZnO bi-composites have shown a low operating temperature (i.e., 275 °C). On the other hand, bare ZnO particles possessed a higher operating temperature. The variation in temperature was mainly due to the inability of the acetone molecules to react with reactive oxygen species on the surface of nanoparticles at low operating temperature.

**Figure 13:**
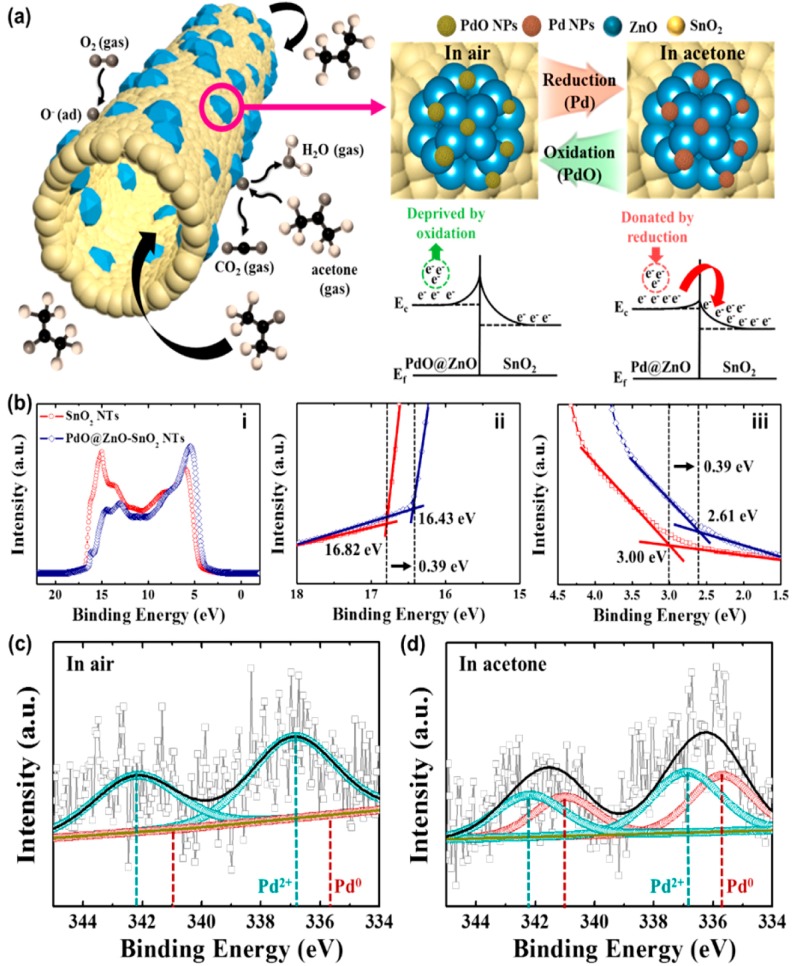
(**a**) Schematic illustration of acetone-sensing mechanism for PdO@ZnO–SnO_2_ nanotubes NTs; (**b**) (i) ultraviolet photoelectron spectroscopy (UPS) spectrum of SnO_2_ NTs and PdO@ZnO–SnO_2_ NTs ((ii) high-binding-energy region and (iii) low-binding-energy region) and ex situ X-ray photoelectron spectroscopy (XPS) analysis using high-resolution spectra of PdO@ZnO–SnO_2_ NTs in the vicinity of Pd 3d (**c**) in air and (**d**) in acetone after a seven-cycle sensing measurement with 5 ppm of acetone at 400 °C. Adapted figure from [96] with permission from copyright (2017), American Chemical Society (Washington, DC, USA).

Koo et al. [96] found that the heterogeneous sensitization of ZnO nanostructures with PdO on the surface of SnO_2_ nanotubes has greatly influenced the catalytic activity of the nanomaterials and enhanced the sensitivity for acetone sensing (Figure 13). The improvement in the activity was primarily interpreted by the formation of n−n type heterojunctions between the PdO@ZnO/SnO_2_. Secondly, the catalytic efficiently was also affected by the presence of PdO and ZnONPs. The presence of differences in the work function further influenced the efficiency of the sensor for acetone molecules. These variations were easily captured by using the ultraviolet photoelectron spectroscopy (UPS) spectrum of surface-functionalized nanotubes of SnO_2_ nanotubes (NTs) with PdO@ZnO under He I radiation with potential of 21.22 eV. The respective cut-off for the binding energy was found to be 16.82 eV for SnO_2_ NTs and 16.43 eV in the presence of PdO@ZnO–SnO_2_ NTs. The presence of high work function for Pd-laden ZnO (5.34 eV) compared to SnO_2_ NTs (4.40 eV) made the movement of the electrons from the conduction band in SnO_2_ to ZnO. As a result, there was a significant shift in the energy toward the Fermi level of ZnO. Such variations caused the bending of conduction bands in an upward direction by the chemisorption of oxygen molecules. As a result, a potential barrier was initiated on the interface of PdO@ZnO/SnO_2_. As an consequence, the baseline resistance value for the PdO@ZnO–SnO_2_ NTs was amplified by ~6 kΩ as compared to bare nanotubes of SnO_2_ NTs. Consequently, the sensing ability of the formed material was found to be higher for acetone.

**Figure 14:**
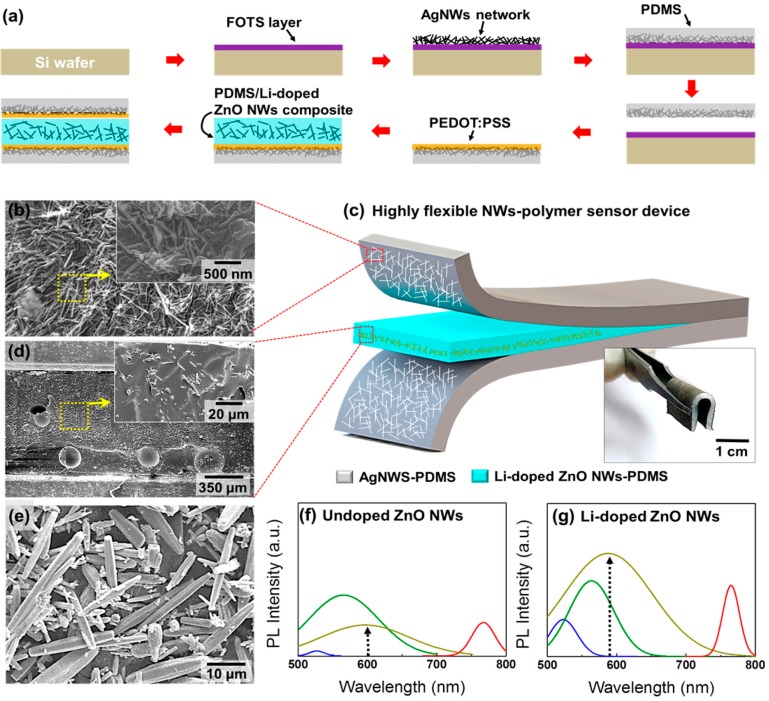
(**a**) Schematic illustration of the sensor fabrication process: composite layer, containing piezoelectric Li-doped ZnO NWs and PDMS, is sandwiched between two poly (3,4-ethylenedioxythiophene) polystyrene sulfonate (PEDOT:PSS)-coated Ag NW electrodes embedded in the PDMS; (**b**) SEM of the AgNW network, which is seamlessly connected on the PDMS surface. The inset shows a magnified view of the Ag NWs on the PDMS; (**c**) Schematic representation of the sensor device consisting of two main parts: resistive and piezoelectric sensing elements. The inset shows the flexibility of the device; (**d**) Cross-section of the device SEM; (**e**) SEM of as-synthesized Li-doped ZnO NWs; (**f**) photoluminescence (PL) spectra of undoped ZnO NWs, and (**g**) Li-doped ZnO NWs. The yellow emission explicitly indicates the Li-doping in ZnO. Adapted figure from [97] with permission from copyright (2017), American Chemical Society (Washington, DC, USA).

In addition, the piezoelectric layer made up of Li-doped ZnO nanowires and PDMS polymer had the ability to produce an effective assimilated structure which provides a dynamic sensing material for the detection of acetone molecules at low concentrations (Figure 14).

These sensors had direct control over the additional motion and enabled the detection of instantaneous motion in the presence of external analyte. By successfully integrating these two elements in sensory devices, the respective sensing limit and sensitivity was managed to a greater extent. The as-prepared sensor displayed excellent mechanical elasticity, strength, and stretching capability, which are quite appropriate for different types of applications involving the investigation of analyte at low working temperature. It was also found that the obtained sensitivity of the sensor was mainly dependent on the surface defects present in the nanostructures. In addition, the surface area of the nanostructures also affected the interaction with the chemical molecules. Post-management during the synthesis of nanoparticles is one of best ways to adjust the defect structures on the surface of the prepared nanomaterials. The calcinations temperature also modulated the amount of defects on the surface of the prepared ZnO particles. In addition, the activation of nanomaterials under low-pressure conditions enhanced the catalytic activity of ZnO nanomaterials by providing a higher mass of oxygen vacancy (VO) defects on the exterior surface of nanoparticles. The agglomeration of nanostructures during synthesis has the tendency to decrease the efficiency of the sensor. Therefore, care was taken to control the size and agglomeration rate and luminescence during the synthesis of ZnO nanomaterials in order to obtain good chemical sensing results.

### 3.7. Other Chemical Sensors Based on ZnO Nanomaterials

Currently, the threat caused by the excessive use of heavy elements is a matter of concern among researchers. These heavy metals cause several types of health-associated illness, and in extreme cases cause death in human beings. There are several types of elements which are required for the proper growth of living beings. Yet, their accretions in living systems beyond tolerable limits can cause severe health predicaments due to their lethal nature. The noxious nature of a heavy metal is mainly associated with its oxidation state, concentration value, composition of the solvent media, and many other factors. Thus, there is an urgent need for a simple process to detect heavy metals. In this regard, ZnO-based nanostructures have had significant importance for providing an effective means of detecting heavy metals with great accuracy and precision. The morphology of ZnO-based nanostructures has a pronounced influence on the sensing ability of these metal ions. It has been ascertained that the nature of morphologies such as nanoflowers, cones, tubes, nails, urchins, prisms, and wires have the ability to influence the prime features of the prepared chemical sensors. These shapes have a direct effect on the limit of detection, sensitivity, reaction time, linear range, working potential, and optical activity of the produced sensor. These morphologies further affect the surface area, width, catalytic activities, electron transport characteristics, and many other properties of the developed sensor.

**Figure 15:**
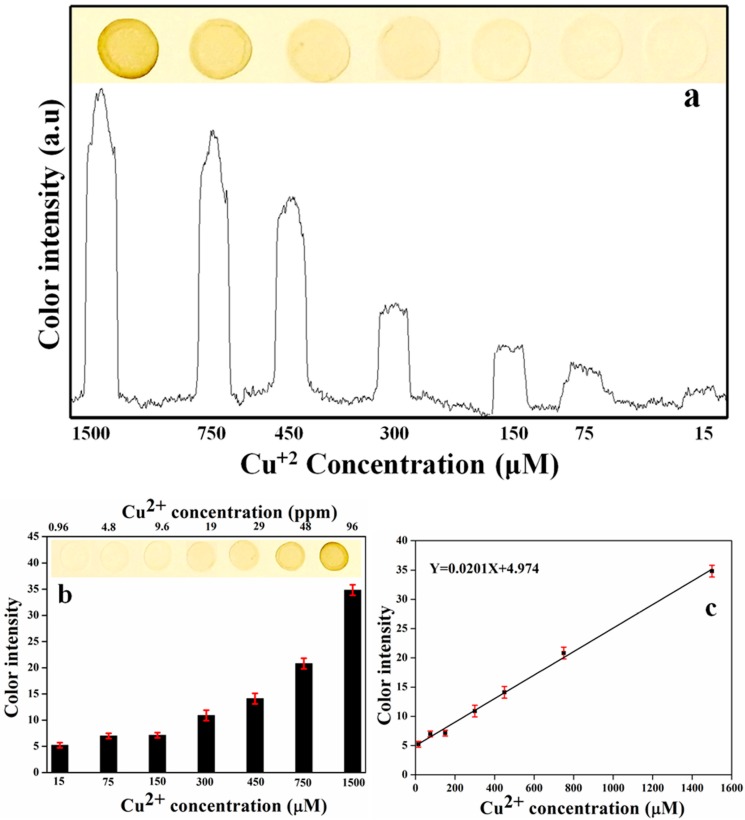
(**a**) Color intensity versus the Cu^2+^ ions concentration obtained by ImageJ with a digital photograph as an inset; (**b**) The color intensity versus the Cu^2+^ ions concentration; and (**c**) The calibration curve of the color intensity at different concentrations of the Cu^2+^ ions. Adapted figure from [103] with permission from copyright (2014), American Chemical Society (Washington, DC, USA).

For instance, Bhanjana et al. [98] used well-characterized ZnO nanoparticles for the analysis of cadmium ions by using the electrochemical method. The advanced redox activities of ZnO nanoparticles provide an effective means for the analysis of these cadmium ions. The measurements were performed by using three electrode systems in which Ag/AgCl acted as reference, platinum wire worked as counter electrode, and the ZnO nanomaterials on the surface of an Au electrode acted as working electrodes, respectively. From the data, it was found that the electrochemical response for cadmium ion was reversible in nature, as CV plots displayed both oxidation and reduction peaks. The presence of ordered defects on the surface of ZnO nanomaterials makes them efficient electrocatalysts for the estimation of cadmium. Moreover, ZnO nanostructures also possess specially exposed reactive sites with advanced electronic configuration and improved electro-catalytic action. As a consequence, charge circulation at the electrode/electrolyte interface is amplified due to the development of an electrical double layer with smallest contact resistance on the interface. In addition, ZnO nanoparticles in combination with Ag nanostructures were also employed for the determination of Pb^2+^ and Cd^2+^ ions in a buffer media with the pH of the reaction media equivalent to 5 [99]. It was found that modified glassy carbon electrodes with ZnO@Ag nanoparticles displayed considerable anodic and cathodic peaks in the presence of the studied metal ions at a scan rate of 50 mV/s. Conversely, no peak was observed for the oxidation of zinc into zinc ions. The small water-soluble quantum dots (QDs) of ZnO nanodots have also had a significant role in the elimination of harmful and toxic chemicals [100]. The generation of the particles was achieved from pomegranate peels. The synthetic process was found to be quite economical and more feasible for the larger-scale synthesis of ZnO nanoparticles. The requirement of low-temperature conditions for the preparation of ZnO nanodots further made the process more economical. The prepared particles displayed good control over the optical and luminescence properties of ZnO, and provided better material for the preparation of chemical sensors for the metal ions. The developed sensor was quite selective for the estimation of Cr^3+^ ions, as it was found that out of different types of metal ions, the corresponding emission from ZnO QDs was found to be highly quenched in presence of Cr^3+^ ions. The effective interaction between the Cr^3+^ and the ZnO QDs had the ability to sense the small amount of ~2 nM of Cr^3+^ ions from the aqueous media. Additionally, a recent approach for the detection of Cu^2+^ in aqueous media was carried out by using glycol-modified ZnO nanoparticles [101]. The detection of metal ions was done by the quenching of fluorescence intensity at 351 nm in the presence of Cu^2+^ ions. These Cu^2+^ ions have the ability to encourage the aggregation of particles, and resulted in the quenching of fluorescence intensity of ZnO nanomaterials. The working range of the formed sensor was 10–200 nM, with a limit of detection of 3.33 nM. The competence of the prepared sensor was fairly high in real water samples from different sources. Ng and co-workers [102] produced a turn-off luminescent sensor for Cu^2+^ ions by using ZnO nanomaterials. The developed sensor showed good control over the selectivity and sensitivity for the metal ions under study, with the limit of detection (LOD) of ~7.68 × 10^−7^ M. The biocomposites of ZnO with ZnS nanostructures have the potential for the estimation of Cu^2+^ ions in aqueous media (Figure 15). The field site detection of metal ions was done by preparing the test papers by using ZnO@ZnS nano materials [103]. In order to investigate the response of the developed paper sensor for the selective sensing of Cu^2+^ ions from aqueous media, the solutions were prepared by using different concentrations (15, 75, 150, 300, 450, 750, and 1500 μM). The pH of the reaction media was kept acidic (pH ~ 4) in nature, with buffer concentration of 10 mM, respectively. For the estimation, around 20 µL of every solution was drop-casted on the paper piece of the developed sensor. The paper was kept for drying for at least 20 min, and respective photographs were taken by using a digital camera (Figure 15).

**Figure 16:**
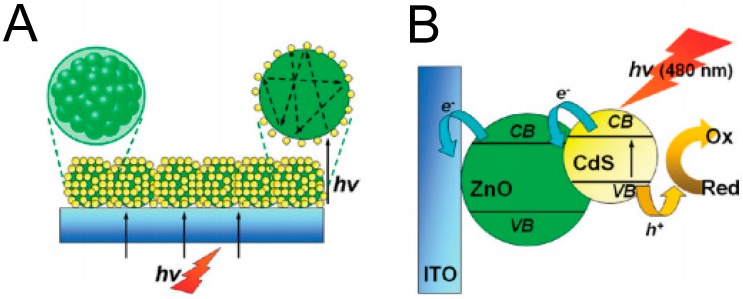
(**A**) Schematic of light scattering occurring on a ZnO/CdS-modified electrode; (**B**) Electron–hole pairs generation, separation, and transfer between ZnO and CdS at a ZnO/CdS-modified electrode for the sensing of Cu^2+^ ions where CB and VB are the conduction and valence band. ITO is Indium tin oxide. Adapted figure from [105] with permission from copyright (2011), American Chemical Society, (Washington, DC, USA).

In separate studies, the surface modification of ZnO nanomaterials with imine moieties had the tendency to detect Co^2+^ ions in aqueous media [104]. Shen et al. [105] employed a hierarchical mixture of ZnO with CdS nanospheres for the selective sensing of Cu^2+^ ions by using the photo-electrochemical method. ZnO nanostructures have the ability to supply an adequate quantity of light scattering centers on the surface of particles for the effective sensing of metal ions. The heterojunctions between the ZnO and CdS materials can present substantial enhancements to light absorption and charge separation. As a consequence, these biocomposites are effective for the enhancement of photocurrent intensity. It was also found that the excessive accumulation of Cu^2+^ ions on the surface CdS solution induces the effective binding of Cu^2+^ with S^2−^ and leads to the reduction of Cu^2+^ to Cu^+^ under illumination and hence detects these ions. The as-formed Cu_x_S on the exterior surface of CdS nanoparticles provides a lower energy level in the particles that acts as an effectual pathway for the recombination of electrons and holes in the band gaps (Figure 16).

The utilization of surface-enhanced Raman spectroscopy (SERS) for the sensing of Hg^2+^ ions from aqueous media was also made possible by using ZnO nanostructures [106]. Hg^2+^ is a highly noxious metal ion which is considered to be a focus in metal ion sensing. The combination of Ag ions with ZnO nanostructures make it more Raman-active and have the ability to effectively bind with Hg ions. These ions are not only detected with these nanostructures, but metal ions are also fully regenerated when external heat treatment is given to the samples. Therefore, it can be said that the ZnO nanostructures have offered the self-cleaning ability of Schottky junctions, and perform as an effective photocatalyst for harmful metal ions. Jacobsson and Edvinsson [107] have studied the characteristic properties of ZnO nanostructures by employing simple and reliable adsorption and fluorescence spectroscopic measurements. The influence of size and band gap of ZnO nanostructures were mainly associated with the available mobile trap states in as-prepared ZnO nanostructures. The luminescence properties used for the estimation of metal ions were controlled by changing the reaction parameters of the system during the particles’ synthesis [108]. The presence of capping agents affected the surface defects on the surface of nanoparticles and further influenced the bioimaging role of as-formed nanoparticles.

## 4. Conclusions and Future Directions

The effective chemical sensors developed by using ZnO-based nanostructures has great potential in environmental remediation. Their effective surface area with biocompatible nature and manageable pore size with controlled morphologies has further boosted their range in the activities related to chemical sensing. The controlled synthesis with optimized band gap has enhanced their role in UV-based metal ion sensors. The optimized photoluminescence properties of ZnO have provided an effective range of chemical sensors for metal ions. The development of next-generation devices with white light-emitting properties makes them effective materials in optoelectronics. The present review meticulously introduced the current advancements of the ZnO nanostructures-supported sensors with a main emphasis on chemical sensors for different analytes. The different operational factors such as effect of size, morphology, and the respective working mechanisms of nano-ZnO-based sensors along with their selectivity and sensitivity were also considered in detail.

## Figures and Tables

**Table 1:** A response comparison of as-fabricated sensors in this work with various ZnO nanostructure-based hydrazine sensors.

Electrode Material	Sensitivity (µA·µM^−1^·cm^−2^)	Limit of Detection (µM)	Linear Range (µM)	Response Time (s)	Ref.
Nano-nails of ZnO	-	0.2	0.1–1.2	<5	[46]
Nanowires of ZnO with high aspect ratio	-	0.0847	0.5–1.2	<5	[47]
Nanorods of ZnO	4.76	2.2	0.2–2.0	<10	[48]
Hierarchical micro/nanostructures of ZnO	0.51	0.25	0.8–200	<3	[49]
Nanorods of ZnO/FTO	0.44	515.7	-	<10	[50]
ZnO nanorods/alloy	4.48	0.2	-	<8	[51]
ZnO nanorods/Single walled carbon nanotube	0.1	0.17	0.5–50	-	[52]
Reduced graphene oxide/ZnO-Au	5.54	0.018	0.05–5	<3	[53]
Cetyl pyridine chloride/ZnO	0.172	24	1000–60,000	-	[54]
ZnO-1/Au/gold electrode ZnO-2/Au/gold electrode ZnO-3/Au/gold electrode	1.70 2.97 1.25	0.25 0.17 0.08	0.8–251 201–851 101–851	<3 <3 <3	[55]
